# Benefits, concerns, and sustainable alternatives to genetically modified crops from a global and Indian perspective

**DOI:** 10.1002/tpg2.70154

**Published:** 2025-12-10

**Authors:** Chittaranjan Kole, Sarita Pandey, Jeshima Khan Yasin, Sujan Mamidi, Abhishek Bohra, Poulami Bhattacharya, Devraj Dhanraj, Gnanasekaran Madhavan, Dinesh Saini, Sayak Ganguli, Bhargavi HA, Sayanti Mandal, Sangita Agarwal, Arumugam Pillai M, Madhugiri Nageswara‐Rao, Swarup K. Chakrabarti, Prakash C. Sharma, Akshay Talukdar, Jogeswar Panigrahi, Manikanda Boopathi N

**Affiliations:** ^1^ Tagore Society for Rural Development Kolkatta India; ^2^ School of Agriculture and Allied Sciences The Neotia University Sarisha West Bengal India; ^3^ Division of Genomic Resources National Bureau of Plant Genetic Resources New Delhi India; ^4^ HudsonAlpha Institute for Biotechnology Huntsville Alabama USA; ^5^ S.V. Agricultural College Tirupathi India; ^6^ ICAR‐Indian Institute of Pulses Research (IIPR) Kanpur India; ^7^ Genetics and Plant Breeding The Neotia University Sarisha West Bengal India; ^8^ The Graduate School ICAR‐Indian Agricultural Research Institute New Delhi India; ^9^ Department of Agronomy Tamil Nadu Agricultural University Coimbatore India; ^10^ Department of Plant and Soil Science Texas Tech University Lubbock Texas USA; ^11^ Postgraduate and Research Department of Biotechnology St. Xavier's College (Autonomous) Kolkata India; ^12^ Division of Vegetable Crops ICAR‐Indian Institute of Horticultural Research Bengaluru India; ^13^ Department of Chemistry and Biochemistry Sharda School of Basic Sciences & Research Sharda University Greater Noida India; ^14^ Department of Applied Science RCC Institute of Information Technology Kolkata India; ^15^ Department of Plant Breeding and Genetics Agricultural College and Research Institute Tamil Nadu Agricultural University Killikulam India; ^16^ USDA‐ARS‐Subtropical Horticulture Research Station Miami Florida USA; ^17^ ICAR‐CPRI, Shimla Kolkata India; ^18^ University School of Biotechnology Guru Gobind Singh Indraprastha University New Delhi India; ^19^ Genetics Division ICAR‐Indian Agricultural Research Institute, Pusa New Delhi India; ^20^ PG Department of Biotechnology Berhampur University Berhampur India; ^21^ Department of Plant Biotechnology, Center for Plant Molecular Biology and Biotechnology Tamil Nadu Agricultural University Coimbatore India

## Abstract

The global population, set to exceed 10 billion by 2050, presents enormous challenges to food, health, nutrition, energy, and environmental security. Plant breeding methods have continuously evolved to develop improved crop varieties to meet these demands. Among the recent developments, genetically modified crops (GMCs) have emerged as a viable option to enhance crop yields, nutritional value, biofuel potential, and climatic adaptability. However, extensive application of GMCs is a very controversial subject due to biosafety issues, environmental impacts, economic viability, and legal considerations. This review presents a critical evaluation of the merits and limitations of GMCs, along with a discussion of available alternative approaches, with particular reference to the Indian context. While GMCs have been developed with increased yields, improved shelf life, reduced pesticide and herbicide use, and improved stress tolerance, potential risks such as health hazards and socioeconomic impacts on smallholding farmers in the developing world cannot be disregarded. Besides, regulatory policies and public perception have a significant influence on the acceptability and commercialization of GMCs, especially in countries like India. The discussion therefore encompasses other sustainable alternatives, including marker‐assisted selection, genomics‐aided breeding, cisgenesis, intragenesis, and stringently regulated gene editing, that embody environment‐friendly approaches to agricultural enhancement. A collective assessment of these techniques is presented in order to examine their prospects for delivering long‐term biosecurity without compromising environmental and human health. By integrating scientific advances, policy environments, and social perceptions, this review aims to present a balanced perspective of GMCs and their role in the future of global agriculture, particularly in the Global South.

## Plain Language Summary

By 2050, the world's population is expected to exceed 10 billion people. This growth will increase the need for food, nutrition, energy, and environmental protection. Genetically modified crops (GMCs) have been developed to help meet these needs by improving crop yields, boosting nutritional value, reducing pesticide use, and helping plants survive under climate stress. This review looks at both the benefits and challenges of using GMCs, with a special focus on India. It also explores newer, more precise crop improvement methods such as marker‐assisted breeding, genomics‐based selection, cisgenesis, and intragenesis. These techniques aim to make farming more productive and sustainable while protecting health and the environment. GMCs have successfully improved yields and reduced chemical use in some crops, such as *Bacillus thuringiensis* cotton. However, public concerns remain about food safety, environmental effects, and how these technologies affect small farmers. India's regulatory system and public opinion continue to shape how such technologies are accepted and used. Emerging gene‐editing tools are seen as safer and more acceptable alternatives when carefully managed.

**Abbreviations**
Bt: *Bacillus thuringiensis*
CRISPR: Clustered Regularly Interspaced Short Palindromic Repeats
DBT: Department of Biotechnology
DMH‐11: Dhara Mustard Hybrid‐11
EC: European Commission
EPA: Environmental Protection Agency
FDA: Food and Drug Administration
GE: genetically engineered
GEAC: Genetic Engineering Appraisal Committee
GM: genetically modified
GMC: genetically modified crop
GMOs: genetically modified organism
GS: genomic selection
ICAR: Indian Council of Agricultural Research
IIRR: Indian Institute of Rice Research Institute
IP: intellectual property
MAS: marker‐assisted selection
NAHEP: National Agricultural Higher Education Project
NIPB: National Institute of Plant Biotechnology
NRCPB: National Research Centre on Plant Biotechnology
SAUs: State Agricultural Universities
SDN: site directed nuclease

## 1 INTRODUCTION: AGRICULTURAL IMPERATIVES AND BREEDING INNOVATIONS

The purpose of human civilization has been inextricably linked with agriculture, and breeding plants has been the key to food security. Breeders have increasingly improvised methods and practiced genetic selection ever since the start of crop domestication to develop varieties capable of keeping up with the continuously mounting demands of a growing world population. This historical process has placed on the market crops with enhanced productivity, nutritional value, and tolerance to environmental stresses. However, human society is currently confronted with an unprecedented demographic crisis: catering to an additional 2 billion people by the year 2050, a challenge made increasingly aggravated by the increasing impact of global warming, which enunciates both abiotic and biotic stresses on agriculture (Kole et al., 2015). Moreover, global warming, for instance, encourages the spread of new plant pathogens, which pose a constant threat to plant yields and food webs (Ristaino et al., 2021).

Plant breeding programs have changed significantly to meet these sophisticated challenges, venturing beyond simple crossbreeding and adopting advanced biotechnological techniques. Of these, genetically modified crops (GMCs) have emerged as a powerful, if contentious, tool to introduce novel characters and accelerate crop improvement. This review attempts to offer an integrated and balanced perspective of GMCs, that is, by describing their anticipated advantages, associated concerns, and the situation with regard to alternative breeding options, with specific allusion to the Indian context.

In doing so, the article begins with the fundamental aims of crop breeding, explaining the historic and modern objectives that are the bedrock of agricultural innovation. It then covers achievements and limitations of conventional breeding, laying the foundations for why modern biotechnologies reached the fore. Following this is a thorough description of molecular breeding, outlining how it differs from genetic engineering as well as its developments. The review subsequently introduces genetically modified organisms (GMOs), chronicling the history behind their creation and how genetic engineering was seen as a solution to agricultural challenges. A significant portion of the section is dedicated to the history of transgenic research in India, detailing government programs, results, and GMC production and regulatory status today. Subsequently, this article offers a critical examination of the commercialization of research outcomes for transgenic crops based on global as well as Indian perspectives, success, limitations, and potential challenges.

A critical part of this review is dedicated to assessing the risk‐benefit ratio of genetic modification, examining issues related to foreign DNA issues, unforeseen health effects, gene flow, loss of biodiversity, increased herbicide use, and the creation of “super weeds” and “super pests.” It also addresses ethical issues and responsibility for future generations. This brings about the subject of biofortification, comparing transgenic and non‐transgenic approaches to nutritional improvement. Finally, the review addresses consumer concerns of genetically modified (GM) food products across various countries and highlights opportunities in sustainable plant breeding and agroecology as valuable alternatives or complements to transgenic approaches toward an integrated approach to long‐term food security and environmental integrity.

**Core ideas**

- Feeding the growing global population will require smarter, more sustainable farming technologies.
- Genetically modified crops (GMCs) offer real benefits but also raise important ethical, environmental, and social questions.
- Modern genome‐editing tools, when properly regulated, can balance innovation with safety.
- Building public trust, strong governance, and farmer participation is essential for the responsible use of crop biotechnology in India and worldwide.

## 2 OBJECTIVES OF CROP BREEDING

The primary objective of crop breeding has traditionally been the enhancement of yield to support a growing world population (Tester & Langridge, 2010). Through the selection and breeding of crops for higher productivity, breeders have traditionally taken advantage of naturally present genetic potential within crop varieties, thereby improving both yield per unit area and overall resource use efficiency without extensive genetic modification (Acquaah, 2012). A further fundamental goal is to increase resistance to biotic stresses, that is, pests and diseases, through the creation of varieties with the desired genetic composition that take advantage of the genetic diversity present in plant germplasm resources, minimizing the use of chemical inputs and ecological effects (Carvalho, 2006). Simultaneously, breeding for tolerance to abiotic stresses such as drought, salinity, and temperature extremes remains relevant, particularly in light of ongoing climate change (Kole et al., 2015; Mittler, 2006). Additionally, improvement of quality traits such as nutritional value and post‐harvest shelf life continues to be a primary breeding goal in order to reduce food losses and enhance consumer acceptance (Akhtar, 2015). Collectively, these strategies aim to achieve yield stability, enhance tolerance to environmental stresses, and improve nutritional value in a sustainable manner, underscoring the importance of harnessing existing crop diversity (Foley et al., 2011).

### 2.1 Conventional breeding—Successes and limitations

Conventional plant breeding relies largely on various methods of selection and the formation of new genetic combinations through sexual hybridization, followed by phenotypic selection of superior individuals and populations. This process has continuously produced improved cultivars, resulting in significant yield enhancement in most economically important crops. Such advances have, specifically, outpaced neo‐Malthusian projections for food production and population growth (Fedoroff, 2010). For example, global cereal yields increased from 1.35 to 8.71 t/ha between 1961 and 2022 (FAO, 2022), largely as a result of conventional breeding and better cultivation practices. The Green Revolution is one such historic example where the identification of dwarf genes in rice and wheat resulted in the development of high‐yielding, non‐lodging varieties (Evenson & Gollin, 2003). Similarly, US maize yields have increased fivefold since 1930 (Duvick, 2005). Despite these advances, conventional breeding has inherent limitations: it depends on favorable genes occurring naturally, takes on average 12–15 years to develop a new cultivar, and can transmit unwanted traits along with desired traits due to linkage drag (Breseghello & Coelho, 2013; Kole & Gupta, 2004).

### 2.2 Molecular breeding—Advances and limitations

The recent advances in molecular breeding have been tremendous, and some of the significant achievements encompass the development of disease‐resistant, drought‐tolerant, and high‐yielding crop varieties (Kole & Gupta, 2004; Varshney et al., 2021). Utilization of marker‐assisted selection (MAS) and other such techniques enables one to map the desirable trait with precision onto linkage maps and thus accelerate breeding activities compared to conventional methods (Boopathi, 2020). For example, molecular breeding has facilitated the creation of rice varieties resistant to bacterial blight, quality protein maize, and wheat with improved resistance to rust diseases (Varshney et al., 2021). Genomic selection (GS) extends these possibilities by predicting the breeding value of plants and animals using genome‐wide markers, which improves the accuracy and efficiency of selecting elite genotypes without the need for large‐scale field testing (Sinha et al., 2021). At this point, it is imperative to distinguish molecular breeding from genetic engineering (transgenic approaches).

Molecular breeding and genetic engineering are distinct approaches in modern crop improvement, differing in both methodology and public perception. Molecular breeding refers to the use of molecular markers to identify, track, and select desirable genes from within a plant's own gene pool or that of sexually compatible relatives. It leverages natural genetic variation and recombination, facilitating precise and accelerated selection without altering the species barrier. It encompasses MAS and marker‐assisted backcrossing, which are widely accepted by regulators and the public, as it aligns closely with conventional breeding principles and avoids introducing foreign DNA. However, molecular breeding is not without limitations. It depends on existing genetic diversity, and its success is constrained when the desired trait is absent in accessible germplasm. The cost and technical demands of genotyping and data analysis can be prohibitive, particularly for resource‐limited breeding programs. Thus, the process remains resource‐intensive, requiring advanced genotyping platforms, robust phenotyping, and long breeding cycles, factors particularly challenging in developing regions. Additionally, many agronomic traits are governed by complex gene networks and environmental interactions, making trait improvement via simple MAS strategies difficult without integrative genomic and phenomic support. Finally, simple MAS strategies may fall short in addressing polygenic traits, necessitating the integration of systems biology and multi‐omics for future breeding success (Boopathi, 2020).

In contrast, genetic engineering involves the direct modification of the genome, often through the insertion of transgenes from unrelated organisms, which can trigger regulatory, ethical, and biosafety concerns as detailed below.

## 3 GENETICALLY MODIFIED ORGANISMS

Genetic engineering is the direct manipulation of an organism's genes using biotechnology. GMOs are organisms whose genomes have been altered in ways that typically cannot occur naturally through normal breeding practices or the natural recombination of genes within a species. These genetic alterations usually involve the transfer of genetic material from unrelated species, bypassing natural reproductive barriers and resulting in the stable integration of foreign DNA sequences into their genomes. These foreign DNA sequences can subsequently be transmitted to future generations according to the principles of heredity.

The history of GMOs dates back to the 1970s, with one of the earliest applications being the production of human insulin. This innovation emerged in response to the limited supply of bovine insulin and effectively addressed a major pharmacological crisis (Ladisch & Kohlmann, 1992).

Initially, GMOs were widely accepted in medicine, agriculture, and everyday applications without significant public scrutiny. However, this perception shifted following non‐food‐related incidents, such as the contamination of blood products with HIV and hepatitis B, which triggered public anxiety about biotechnology. These concerns were further intensified by the emergence of “mad cow disease” (Finucane, 2002). Although GMOs were not directly responsible for these events, the public began to associate genetic engineering with potential health risks, leading to growing skepticism and widespread concern about GMOs (Trkulja et al., 2014).

### 3.1 Benefits of genetic engineering in crop plants

Investment in GMCs is considered essential in order to meet the demands of a growing world population amidst a period of increasing environmental limitations. While conventional germplasm sources give valuable direction to crop improvement, they may not always offer the pace and scale that are required for modern agricultural needs. GMCs hold potential solutions for addressing nutritional deficiency, environmental sustainability, and food security issues, thus offering in most instances what cannot be achieved with conventional means alone within a comparable time frame (Yadav et al., 2024). As science continues to advance, GMCs will continue to be crucial to improving food security and resilience to global challenges. Over the past few decades, GMCs have seen increasing adoption across the world. In 2024, global GM crop area reached approximately 209.8 million ha, marking a 1.9% increase over the previous year (https://www.isaaa.org/gmapprovaldatabase/default.asp). That global scale underlines both the promise and the controversies of GM technologies. India is a critical country to examine in the context of GM and gene‐editing technologies. Exploring India provides both a concrete and representative region for assessing how genetic technologies can be integrated, regulated, and socially accepted in smallholder‐dominated farming systems. The lessons drawn from India are likely to resonate with many other countries grappling with similar tensions between innovation, regulation, and equitable agricultural development.

**FIGURE 1:**
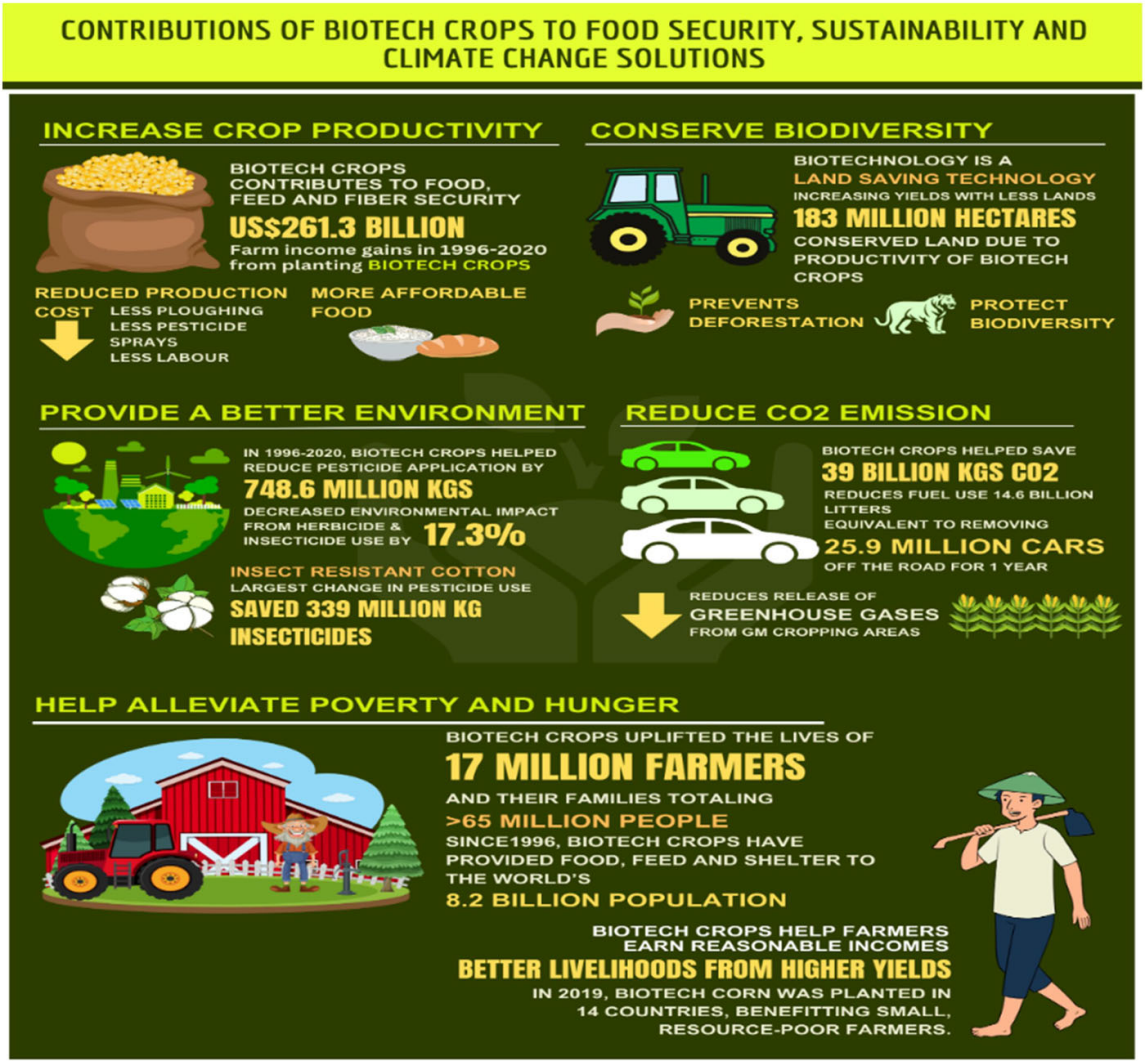
Contribution of genetically modified crops for food security, sustainability, and climate change (data curated from https://www.isaaa.org/gmapprovaldatabase).

GMCs have contributed significantly toward food security, sustainability, and climate change mitigation (Figure 1) by (i) adding agricultural output by $261.3 billion, which translates to an average income increase of $112 per ha. (ii) Promoting environmental sustainability through avoiding 748.6 million kg of active ingredient pesticides released into the environment. (iii) Preventing the emission of 39 billion kg of CO_2_, which amounts to removing 25.9 million cars from the road for a year. (iv) Helping in poverty reduction by improving the livelihood of over 17 million small farmers and their families, cumulatively benefiting over 65 million individuals from economically disadvantaged groups (James, 2011).

**FIGURE 2:**
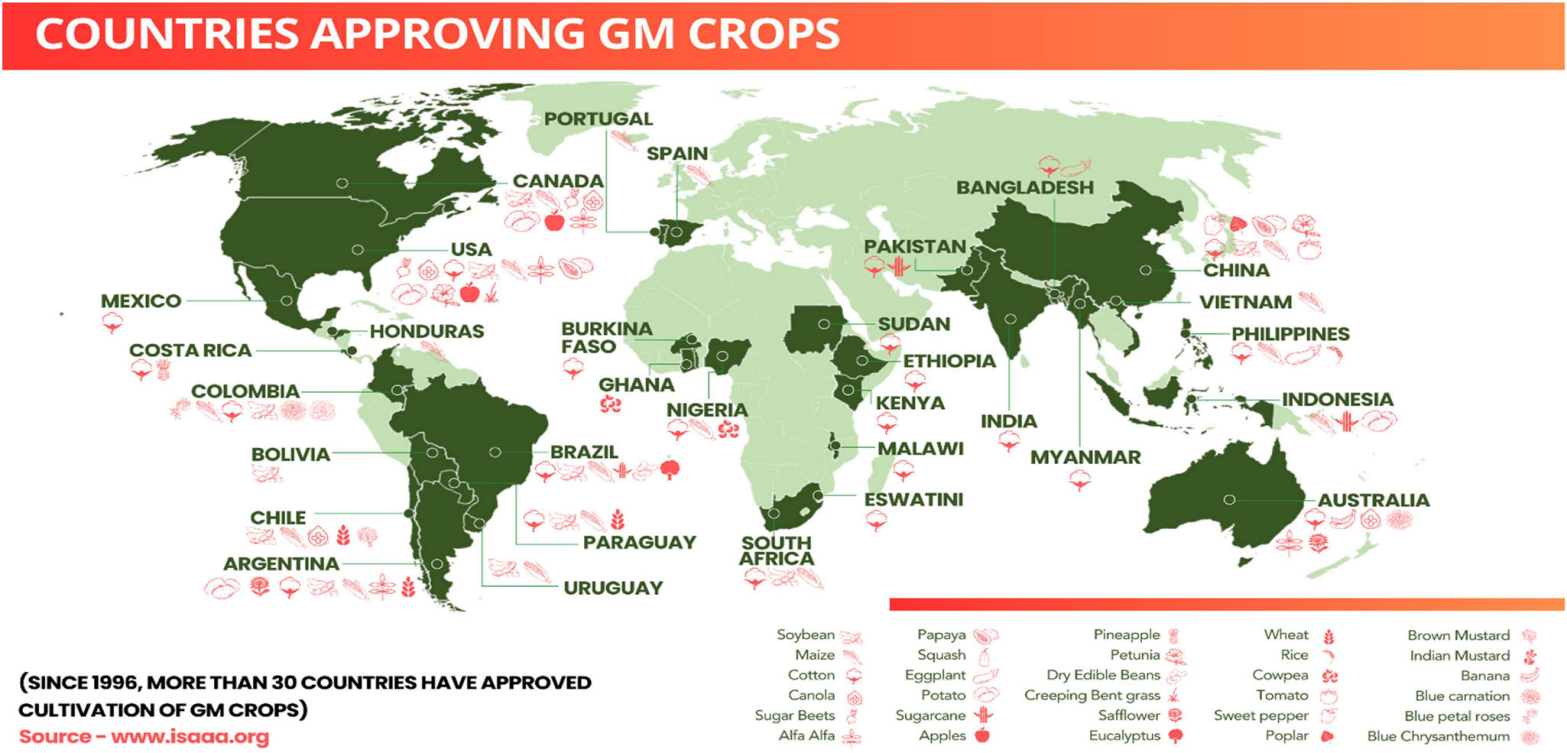
Countries approved GM crops in the last 28 years from 1996 to 2024 (data curated from https://www.isaaa.org/gmapprovaldatabase).

The Flavr Savr tomato was the first GMC to be approved in the United States by the Food and Drug Administration (FDA). This tomato, developed by the California company Calgene, was approved on May 18, 1994, with a foreign gene that would extend its post‐harvest shelf life. Since GMC commercialization began in 1996, their cultivation across the globe has increased progressively in more than 30 countries over the past 28 years (Figure 2).

Understanding global patterns of GM crop adoption can provide valuable insights into the potential economic and environmental benefits for India. Countries such as the United States, Brazil, Argentina, and Canada collectively cultivate over 190 million ha of GM crops, demonstrating measurable gains. Comparative assessments of these experiences help estimate the possible outcomes (such as enhanced farm profitability, lower greenhouse gas emissions, and decreased agrochemical dependence) if similar technologies were adapted under Indian agroecological and socioeconomic conditions.

Notably, the *Bacillus thuringiensis* (*Bt*) *brinjal* case in Bangladesh offers a compelling regional example of the practical success of GMCs. Since its approval in 2013, *Bt brinjal* has significantly reduced pesticide applications, increased farmer incomes, and achieved high consumer acceptance in a context that closely resembles India's agricultural and cultural landscape. Including such evidence from a neighboring country with comparable production systems and dietary habits would strengthen the rationale for a more informed, context‐specific evaluation of GMC adoption and regulatory reforms in India.

## 4 HISTORY OF TRANSGENIC RESEARCH IN INDIA AND ITS KEY OUTCOMES

**FIGURE 3:**
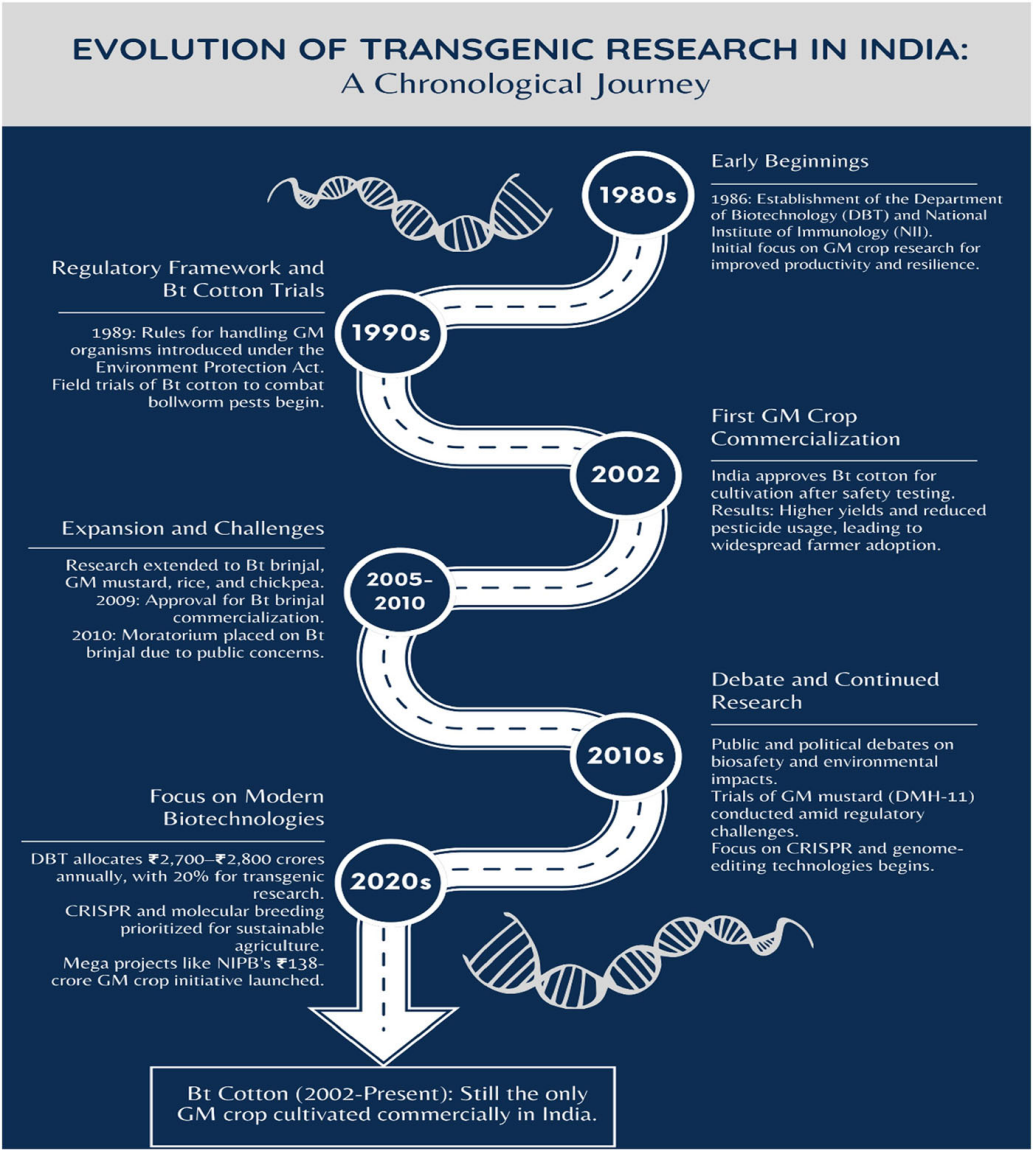
Chronological illustration of the evolution of transgenic research in India.

India, as the world's most populous developing nation with 44% of its labor force employed in agriculture, serves as a critical case study for understanding the status and impact of GMCs. An examination of India's experience provides astute observations of the benefits, challenges, and alternative strategies for other nations. India's venture in transgenic research started in the late 1980s (Ghosh, 1997), driven by the necessity to boost agricultural productivity through the potential of transgenic technologies. Figure 3 illustrates the chronology of significant milestones and events in the history of India's transgenic research.

- 1980s—Beginnings: India began moving into genetic engineering and biotechnology in the 1980s with the establishment of research centers such as the National Institute of Immunology (NII) and the Department of Biotechnology (DBT) in 1986 (Ghose & Bisaria, 2000).
- 1990s—Bt cotton trials and regulatory regime: India started field trials of Bt cotton, which is GM to be resistant to bollworm, a significant pest of cotton, during the 1990s. During more or less the same period, the Government of India notified the “Rules for the Manufacture, Use, Import, Export, and Storage of Hazardous Microorganisms, Genetically Engineered Organisms or Cells” in 1989 under the Environment Protection Act, providing a basic regulatory framework for GM research (Shiva et al., 1999).
- 2002—Commercialization of Bt cotton: India approved the commercial growth of Bt cotton in 2002, the first GM crop grown legally in India. Approval followed rigorous environmental and health safety trials by the Genetic Engineering Appraisal Committee (GEAC). Bt cotton subsequently led to significant yield gains in cotton and reductions in pesticide use, leading to extensive farmer adoption (Karihaloo & Kumar, 2009).
- 2005–2010—Expansion and regulatory challenges: Following the success of Bt cotton, transgenic research expanded to other crops such as Bt brinjal (eggplant), rice, and mustard. Bt brinjal was cleared for commercialization in 2009. However, due to public and political concerns regarding biosafety and long‐term impacts, a moratorium on release of Bt brinjal was announced in 2010 (Rao, 2010).
- 2010‐Present—Continuing research amidst public debate: India has adopted a cautious approach toward the commercialization of GMCs since the moratorium on Bt brinjal. Despite this, active research continues in both public and private institutions. Efforts have extended to crops like GM mustard, which has undergone field trials but faced regulatory and policy challenges (Jayaraman, 2017). The discourse around GM crops remains highly contentious, with ongoing public debate centered on biosafety, ethics, environmental implications, and intellectual property (IP) rights.

To date, Bt cotton is the only GMC that is cultivated commercially in India. Research is, however, ongoing on transgenic mustard, rice, tomato, cotton, banana, and chickpea. Additionally, the discovery of genome‐editing technologies such as Clustered Regularly Interspaced Short Palindromic Repeats (CRISPR) has also opened up new avenues for India's biotechnology sector with the potential of bypassing some controversies associated with conventional transgenic methods.

### 4.1 Key outcomes of transgenic research in India

**FIGURE 4:**
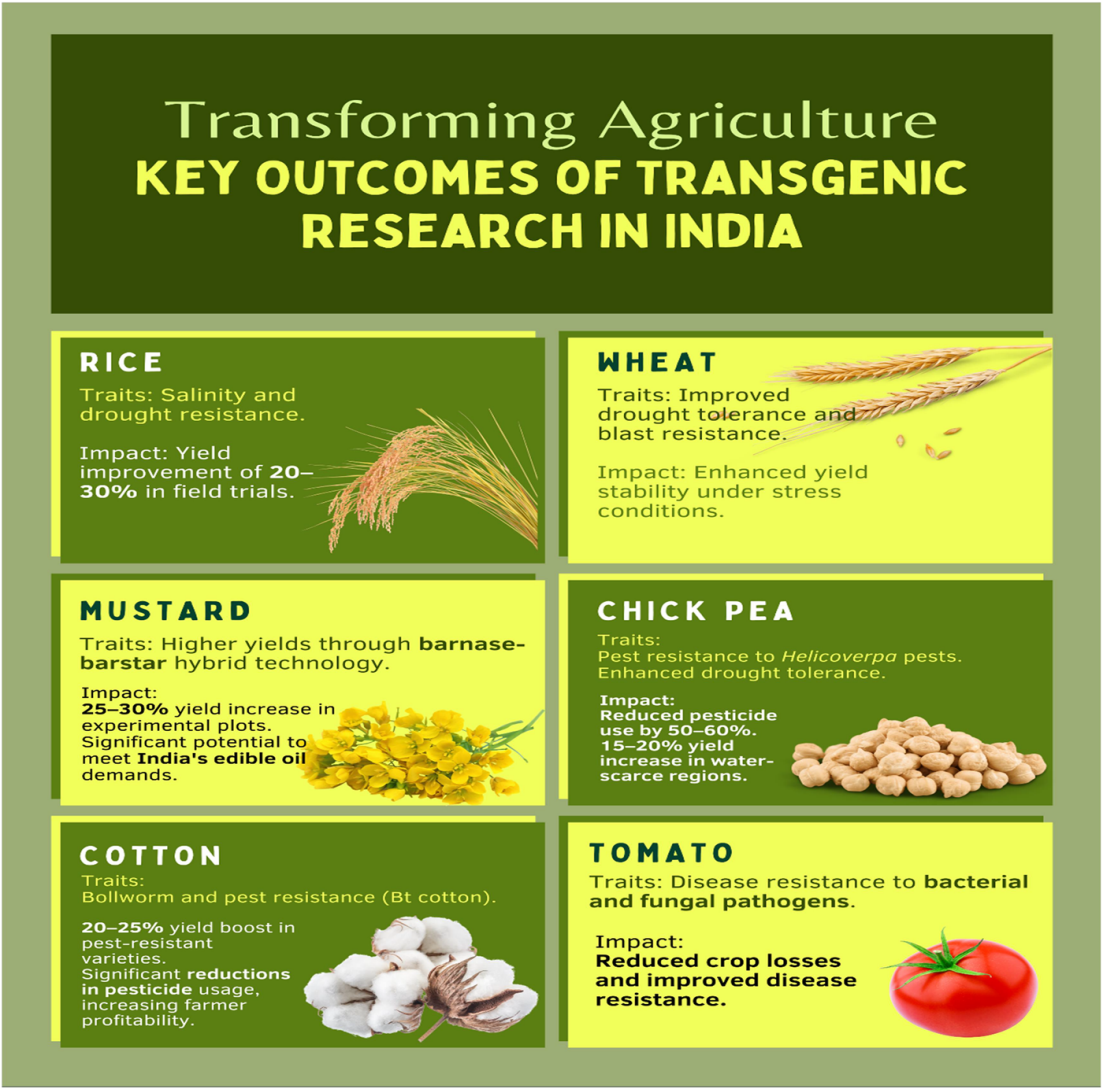
Key outcome of transgenic research in India.

Transgenic research in India has been focused on staple and economically important crops such as rice (*Oryza sativa*), wheat (*Triticum aestivum*), chickpea (*Cicer arietinum*), mustard (*Brassica juncea*), cotton (*Gossypium hirsutum*), pigeon pea (*Cajanus cajan*), and tomato (*Solanum lycopersicum*). These crops were targeted for the development of traits such as pest and disease resistance, productivity enhancement, nutritional quality, herbicide resistance, and abiotic stress tolerance (e.g., drought, salinity, and temperature extremes; Figure 4).

*Quantified progress in transgenic research*: Indian transgenic research has yielded encouraging results across several crops (Figure 4), though most remain at the experimental or trial stage (Akhatar et al., 2025; Dua et al., 2005; Mandaokar et al., 2000; Nagaraj et al., 2024; Shrawat & Armstrong, 2018; Tech Explorist, 2018) and have yet to reach commercial cultivation.

### 4.2 Government funding for transgenic crop research

A brief overview of the funding history of transgenic research in India illustrates the evolution of government support for GMC:

- 1980s—Initial investments: In the late 1980s, the Indian Council of Agricultural Research (ICAR) and the DBT began investing in biotechnology infrastructure and early transgenic research studies. Budget allocations were made for the establishment of research centers and investment in early‐stage transgenic programs (Reid & Ramani, 2012).
- 1990s—Expansion and increased focus: As the interest in GMCs increased, DBT and ICAR enhanced funding for transgenic research. DBT's annual budget increased regularly to ₹200–300 crores ($25–37 million) in the late 1990s (James, 2011). There was increased investment in infrastructure; for example, the National Research Centre on Plant Biotechnology (NRCPB) in New Delhi was established under the auspices of ICAR, and the first field trials of Bt cotton were conducted.
- 2000s—Commercial GMCs gain momentum: Following the clearance of Bt cotton in 2002, investment in transgenic research grew considerably. DBT and ICAR invested approximately ₹400–500 crores ($50–62 million) by the mid‐2000s to allow GM research to extend to other crops like brinjal, rice, and mustard. ICAR's total budget also grew noticeably during the period, with approximately 10%–15% being used for biotechnology and transgenic research.
- 2010s—Focus on regulatory and safety research: Public and environmental pressure and the 2010 moratorium on Bt brinjal caused a rethink in priorities for funding in biosafety research, regulatory research, and further strengthening of trials of transgenic crops. DBT's budget had reached over ₹1000 crores ($125 million) per year by the mid‐2010s, and a very significant proportion was being spent on transgenic research, regulatory biosafety research, and training programs. ICAR's annual budget also witnessed steady increase, reaching approximately ₹7000–₹8000 crores ($875 million–1 billion) by 2015, with a special provision for biotechnology and transgenic research work under the National Agricultural Science Fund (NASF) and All India Coordinated Research Projects (AICRPs) (Sources: ICAR Budget Book 2014–2015 https://icar.org.in/en/icar‐budget‐book‐2014‐15; ICAR Budget Book 2015–2016 https://icar.org.in/icar‐budget‐book‐2015‐16‐0).
- 2020s—Continued focus on new biotechnologies: A significant portion of ICAR's yearly budget is being allocated to new‐generation biotechnologies, including CRISPR and molecular breeding, in addition to transgenics.

Importantly, transformative agriculture initiatives such as Biotech‐KISAN and the National Agricultural Higher Education Project (NAHEP) have seen growing investment, signalling robust government support for integrating advanced genetic technologies into sustainable agriculture. Biotech‐KISAN, a farmer‐centric biotechnology innovation programme under the DBT, has mobilized approximately ₹310 crore ($35 million) over recent years to empower scientists and farmers through regional hubs, entrepreneurship, and applied biotech solutions (https://dbtindia.gov.in/dbt‐press/during‐last‐three‐years‐approximately‐rs‐310‐crores‐has‐been‐invested‐supporting‐use).

Meanwhile, NAHEP, launched in 2017 as a joint effort of ICAR and the World Bank (totalling $165 million or roughly ₹1100 crore), enhances infrastructure, curriculum, and research capability across agricultural universities, including investments in Centres for Advanced Agricultural Science and Technology (CAAST) to support molecular breeding and genomics innovation (https://icar.gov.in/node/10281).

Furthermore, ICAR's National Institute for Plant Biotechnology (formerly NRCPB) has spearheaded a major DBT‐funded project (approximately ₹138 crore (∼$15 million) during 2023–2024) focused on gene discovery, molecular breeding, and transgenic line development to advance crop productivity and resilience. This initiative aligns with India's broader food‐security mandate by integrating cutting‐edge genetic tools into varietal improvement pipelines (https://icar‐nipb.res.in).

### 4.3 Selection of crop (commodity‐wise) for genetic engineering: The case for avoiding food crops

The debate around the adoption of genetically engineered (GE) crops is multifaceted, involving scientific, economic, and societal considerations. The European and Indian experiences with GE crops illustrate why food crops might be better avoided in favor of those intended for commercial or industrial purposes.

The Amflora potato, developed by Badische Anilin‐ und Soda‐Fabrik (BASF) for high amylopectin starch production, was the first GE crop authorized in the European Union (EU). Approved in 2010, it was not intended for food consumption but for industrial applications, with its by‐products permissible as animal feed. The promise was to optimize production processes, saving on raw materials, energy, water, and oil‐based chemicals (Piccioli, 2010; BASF, 2012). Despite these benefits, public resistance to GMOs, even for industrial purposes, led to its market withdrawal in 2012 (Greenpeace, 2012). This resistance stemmed from widespread concern over potential health and environmental risks associated with GMOs, overshadowing the economic advantages (Abdallah, 2010; GMO Compass, 2012).

Bt cotton, engineered to resist certain pests, was introduced in India in the early 2000s and initially seemed promising (James, 2011). However, over time, high seed costs and pest resistance issues led to economic challenges for farmers (Kranthi & Stone, 2020). While Bt cotton did reduce pesticide use initially, subsequent pest resistance nullified these gains, leading to a situation where farmers did not experience the anticipated benefits (Qaim, 2009; Stone, 2011). This highlights the complexities and potential unintended consequences of adopting GE crops, particularly in diverse agricultural environments.

#### 4.3.1 Why avoid GE in food crops?

The European experience with the Amflora potato underscores the critical role of public perception. Despite its industrial focus, the general unease with GMOs affected its market viability (Gaskell et al., 2000). Similarly, in India, the initial acceptance of Bt cotton waned as farmers faced economic and agronomic challenges (Kranthi & Stone, 2020; Stone, 2011). While extensive research indicates limited direct health risks from consuming GE foods, long‐term impacts remain uncertain (Domingo & Bordonaba, 2011; Tsatsakis et al., 2017). Environmental concerns, such as gene flow to non‐GE crops and the evolution of resistant pests, further complicate the scenario (Snow et al., 2005).

The economic benefits of GE crops for farmers are also not always straightforward. High seed costs and the potential for pest resistance can lead to economic instability. Moreover, the lack of consumer demand for GE food crops can limit market opportunities, as seen with both Amflora and Bt cotton (Phipps & Park, 2002; Qaim & Kouser, 2013).

The discussion on GE crops should be balanced, recognizing both the potential benefits and the inherent risks. While GE crops like Amflora and Bt cotton have shown promise, their challenges highlight the need for careful consideration of the specific crop and trait being engineered. For food crops, in particular, the risks and public resistance often outweigh the benefits. Therefore, focusing on GE for industrial or commercial crops might be a more viable strategy, allowing the exploration of GE benefits without the heightened scrutiny and resistance associated with food crops.

### 4.4 Selection of crop (botany‐wise) for genetic engineering: The case against high cross‐pollination crops

The selection of crops for genetic engineering must consider the plant's reproductive biology and the potential for gene flow to wild relatives or non‐GE crops. Crops with high cross‐pollination rates pose significant challenges due to the risk of gene flow, which can lead to unintended environmental and agronomic consequences. The case of GM mustard in India exemplifies these issues.

Cross‐pollination is a primary mechanism for gene flow and is particularly concerning in crops with high cross‐pollination rates like mustard, a widely grown oilseed crop in India (Snow, 2002). India has small land holdings, with an average farm size of about 1.08 ha (https://agcensus.da.gov.in/chartin.html). This fragmented landholding pattern exacerbates the risk of gene flow, as GE crops are often planted near non‐GE crops. The introduction of GM mustard in India has been controversial due to these concerns.

Dhara Mustard Hybrid‐11 (DMH‐11), a GE mustard variety developed by the University of Delhi, uses the barnase‐barstar system to create hybrid vigor. It was designed to increase mustard availability in India and reduce dependence on imports of edible oils. The approval process for GM mustard in India faced delays due to concerns about its potential harm to biodiversity. Small farmers worried about the probable economic consequences of contamination and market rejection of their crops. The lack of clear regulations and compensation mechanisms further heightened their anxieties, particularly regarding the financial burden of adopting new cultivation practices (Munshi et al., 2025).

After its introduction, several issues have been raised. Mustard (*Brassica juncea*) has several wild relatives in India, increasing the risk of transgene escape potentially leading to the creation of “superweeds” (Warwick et al., 2009). Cross‐pollination with non‐GE mustard could result in the unintended spread of transgenes, affecting the purity of non‐GE seed stocks. This has significant implications for organic farming and exports, where non‐GE certification is essential.

The case of GM mustard in India highlights a critical concern in the development of GE crops in cross‐pollinated crops. Similarly, corn (*Zea mays* L.) is a wind‐pollinated crop with some potential for cross‐pollination with wild relatives like teosinte. Unintended transfer of engineered genes, such as herbicide resistance, could create herbicide‐tolerant weeds, making weed control more difficult (Devos et al., 2008). Cotton (*Gossypium* spp.) is another example of a wind‐pollinated crop with wild relatives in some regions. Cross‐pollination between GE cotton and wild relatives could lead to the spread of transgenes beyond intended areas. Rice (*Oryza sativa* L.) is primarily self‐pollinating, but some cross‐pollination can occur with weedy red rice (*Oryza sativa* L. *spontanea*). Gene flow from herbicide‐resistant GE rice to red rice could create problematic herbicide‐resistant weeds, impacting rice production significantly (Lu & Yang, 2009).

Focusing on crops with lower cross‐pollination rates and implementing robust containment strategies can help mitigate these risks and ensure that the benefits of GE technology are realized without compromising environmental and agronomic sustainability.

### 4.5 Herbicide resistance in GE crops: Not a boon for labor‐abundant India (focus on mustard)

While herbicide resistance is a common trait introduced in GE crops (corn, cotton, soybean, canola, sugarbeet), its suitability for all countries, particularly those with abundant labor, needs careful consideration. India, with its large workforce, provides a prime example of why herbicide resistance might not be the most desirable breeding objective (Green, 2012; Kniss, 2018).

India has a vast agricultural labor force, estimated at over 400 million according to the 2011 Census of India. Widespread adoption of herbicide‐resistant GE crops could lead to a decrease in weeding labor, potentially causing unemployment and economic hardship in rural communities (Sims et al., 2018). This impact is particularly critical given the high dependence of rural populations on agricultural employment.

Overreliance on herbicides with herbicide‐resistant GE crops can lead to the evolution of resistant weeds. This phenomenon creates a dangerous cycle, requiring even stronger herbicides and posing potential environmental risks such as soil degradation and water contamination (Devos et al., 2009). This has been observed in other regions where herbicide‐resistant crops are prevalent, leading to increased costs and environmental concerns. Increased herbicide use associated with GE crops can also harm long‐term agricultural sustainability. The environmental consequences, including soil degradation and water pollution, can undermine the ecological balance and lead to adverse effects on biodiversity (K. Kumar et al., 2020).

The development of herbicide‐resistant GM mustard in India exemplifies the potential drawbacks of this technology. Mustard cultivation in India is already labor‐intensive due to the close planting patterns used to optimize yields (Kaur & Kaur, 2017). The introduction of herbicide‐resistant GM mustard might not significantly reduce labor requirements, making the economic benefits questionable. If the expected labor savings do not materialize, the socioeconomic impact could be less favorable than anticipated.

For India, developing GE mustard varieties with traits like resistance to diseases such as *Alternaria* blight or improved yields might be more beneficial. These traits could enhance overall productivity without displacing labor or jeopardizing environmental sustainability (Poveda et al., 2020).

### 4.6 GMCs and smallholding farmers

The impact of GMCs on smallholder farmers remains a subject of active debate, balancing potential economic gains against concerns of equity and inclusion. In India and across the Global South, smallholders constitute over 80% of the farming population, making the socioeconomic implications of GMC adoption particularly significant. However, GMC benefits were unevenly distributed: larger and better‐connected farmers adopted technologies earlier and reaped greater economic returns, while resource‐poor smallholders often faced higher seed costs, limited access to extension services, and vulnerability to market volatility.

Preliminary socioeconomic assessments reveal that justice and inclusivity in GMC deployment depend on access to affordable seeds, transparent regulatory oversight, and institutional support for marginalized farmers. Gender dimensions are also critical, as women farmers frequently lack decision‐making power in seed choice and benefit distribution. Ensuring inclusivity requires public‐sector participation, open‐source biotechnology platforms, and community‐based seed systems that reduce dependency on proprietary technologies. Emerging genome‐editing tools, such as CRISPR, offer new opportunities to democratize innovation, allowing public research institutions to develop locally adapted, smallholder‐focused varieties without the IP barriers that often accompany first‐generation GM crops. When designed within equitable frameworks, GMCs can serve as instruments of technological justice, empowering smallholders rather than excluding them.

Particularly, the socio‐ethical dimensions of GMCs are deeply intertwined with cultural values, traditional farming systems, and public trust in science and governance. Resistance to GM crops often stems not only from scientific uncertainty but also from perceptions of external control over seeds, food, and livelihoods. For millions of smallholders, seeds are part of cultural heritage and community identity; the introduction of patented or corporate‐controlled GMCs is viewed as a potential threat to seed sovereignty and traditional ecological knowledge. Ethical concerns extend to issues of biosafety, intergenerational impacts, biodiversity conservation, and farmers’ rights, especially under the Biological Diversity Act and the Protection of Plant Varieties and Farmers’ Rights (PPV&FR) Act.

Public perception of GM technologies in India remains cautious and fragmented. While Bt cotton has achieved widespread adoption, controversies around Bt brinjal and GM mustard highlight persistent distrust and regulatory hesitancy. Civil society organizations, farmer unions, and environmental groups have often framed GMCs as symbols of corporate dependency and ecological risk. At the same time, the scientific community advocates for evidence‐based policy and transparent risk communication to bridge the gap between innovation and societal acceptance.

Cultural acceptance of GMC in India therefore requires more than technical validation: it depends on ethical engagement, participatory decision‐making, and respect for local values and food traditions. A socially inclusive path forward would combine scientific rigor with community dialogue, ensuring that GMCs are deployed transparently, ethically, and in ways that align with India's agrarian realities, food culture, and sustainability goals.

### 4.7 Strategic deployment of next‐generation innovations in India

Building on decades of progress in conventional breeding and genetic modification, recent years have witnessed an increasing move toward more precise and targeted approaches to crop improvement. Against the backdrop of growing global issues such as climate change, food insecurity, and pest pressure, the use of new biotechnological tools has become imperative to combat these issues. Precision genome editing technologies, driven by designed nucleases such as CRISPR/Cas9, are being exploited by a number of institutes on a vast array of crops, which include rice and mustard (https://innovativegenomics.org/news/crispr‐plant‐biotechnology‐india/). The focus is on modifying yield enhancement and stress tolerance genes (Chowdhury & Gargate, 2021).

Mega‐projects in India have made significant advances in the enhancement of key agronomic traits in different crops, with the involvement of various ICAR institutes such as NBPGR (National Bureau of Plant Genetic Resources), the Indian Agricultural Research Institute (IARI), the Indian Institute of Rice Research (IIRR), and the National Institute of Plant Biotechnology (NIPB) besides state agricultural universities (SAUs) (https://www.nature.com/articles/d44151‐025‐00078‐2). These advances, spanning transgenic as well as genome editing research, have reported promising results, particularly in staple crops like wheat, rice, mustard, and chickpea, and have resulted in quantitative economic product increases. These institutions remain dedicated to addressing critical problems in agriculture, including food security, climate resilience, and sustainable agriculture. Key focus areas of these projects include (i) refinement of existing genetic technologies, (ii) genome editing using CRISPR and base editing, (iii) trait stacking for multiple desirable characteristics, (iv) large‐scale field evaluations and biosafety assessments, and (v) development of improved crop varieties.

## 5 COMMERCIALIZATION OF PUBLIC SECTOR RESEARCH OUTPUTS IN TRANSGENIC CROPS: AN INDIAN AND GLOBAL PERSPECTIVE

Commercialization of transgenic crops presents unique challenges and opportunities globally. Indian and international public research organizations have been at the forefront in the research and development of GM crops. In this section, the status of commercialization of public research outputs for transgenic crops and their contributions, challenges, and success in India and elsewhere in the world are presented.

### 5.1 Role of public research institutions in transgenic crop development

Indian public institutions, including ICAR‐NIPB, ICAR‐IARI, and other institutions under DBT and the Council for Scientific and Industrial Research (CSIR), and SAUs, have been involved in GM crop development. India's commercialization of Bt cotton, in which ICAR played an important role, is a success story of public‐private partnership. Although transgenic rice and wheat for traits like drought tolerance, pest resistance, and higher yield are still being developed at public research institutes, these have yet to be commercialized in India due to biosafety and regulatory concerns.

At the international level, CGIAR centers such as the International Maize and Wheat Improvement Center (CIMMYT) in Mexico and the International Rice Research Institute (IRRI) in the Philippines have been at the forefront of developing transgenic lines of important staple crops such as rice, wheat, and maize. China has also invested heavily in public sector research on GM rice and cotton, leading to the commercialization of insect‐resistant rice and Bt cotton. Public universities and USDA‐affiliated research organizations in the United States have developed GMCs such as corn, soybean, and alfalfa, a number of which have been commercialized in collaboration with private industry (James, 2011).

### 5.2 Effect and measured success of commercialized transgenic crops

The launch of Bt cotton in India in 2002 continues to be a landmark success in the promotion of transgenic crops. Bt cotton, a product of public and private sector collaboration, has tremendously raised India's cotton production, rendering India among the largest exporters of cotton (K. Kumar et al., 2020).

Beyond India, public research in collaboration with the private industry in the United States has commercialized GMCs such as corn, soybean, canola, and alfalfa. These collaborations speak to the contribution that public research institutions make to innovation and the development of new traits, with commercialization often involving private sector involvement. In China, the commercialization of GMCs, in this case Bt cotton and insect‐resistant rice, has resulted in better livelihood. Similarly, public sector research in South Africa has led to the commercialization of GM cotton and maize, which has contributed to increased productivity, especially among smallholder farmers (Lubieniechi et al., 2025; Miller, 2025; Mou et al., 2025).

Bt cotton commercialization in India has had a significant economic impact, with millions of farmers benefiting from improved quality lint production. It is reported that Bt cotton has doubled India's cotton production since its introduction (Choudhary & Gaur, 2010). In the United States, Brazil, and Argentina, GMCs in maize, soybean, and cotton have brought billions of dollars in economic benefits to large‐scale commercial farming as well as smallholding farmers. GMCs such as Bt cotton and Bt maize have resulted in the lowering of pesticide application worldwide. Pesticide application on cotton crops in India has come down by about 50% since Bt cotton was introduced (Peshin et al., 2014). Drought tolerance is one trait that has been included in GMCs through public research and can help decrease water use in agriculture significantly, although most of these varieties are in the trial stage.

### 5.3 Barriers to GMC commercialization

Approval of GMCs in India is done by the GEAC. Though there has been advancement in public research, many transgenic crops have faced prolonged regulatory approval due to environmental and health safety concerns. Globally, European nations have stringent regulations on the commercialization of GMCs, which limit the market for publicly developed transgenic varieties. Negative public sentiment regarding GMCs is a significant barrier in the majority of countries. In India, while Bt cotton has been successful, concerns over environmental and health impacts, cross‐pollination, and corporate control over seed markets have delayed the approval of other transgenic crops. In Europe, strong public opposition to GMCs has resulted in limited commercialization despite advances in public research. Public research institutions are frequently confronted with difficulties in commercializing their technologies due to IP constraints, particularly when private biotech companies hold crucial technologies (e.g., specific genes or transformation methods). Moreover, rigorous biosafety policies, such as on‐field testing and environmental release, may hinder commercialization even after successful development (Lubieniechi et al., 2025).

## 6 ASSESSING THE RISK‐BENEFIT BALANCE OF GMCS

Since there is quite a lot of concern for the environmental and health impacts of GM crops, the benefit offered by any GM variety or hybrid will need to be large enough to decisively override these risks. A risk‐benefit analysis with a great deal of care, considering potential benefits as well as intrinsic hazards, is crucially necessary, and they were elaborated hereunder.

### 6.1 Low yield of GMCs

A GMC developed for a specific breeding objective is generally expected to demonstrate a significant yield advantage (Klümper & Qaim, 2014). A recent case in point is the DMH‐11. According to ICAR trials, DMH‐11 exhibited a 28% higher yield compared to its parent variety, Varuna, and a 37% increase over zonal checks. However, it is important to note that available data do not indicate that the yield of DMH‐11 surpasses that of conventionally bred hybrids currently on the market under comparable conditions (GRAIN, 2023; Jayaraman, 2017). This example highlights that while GMCs may offer targeted advantages, such as hybridization efficiency or stress resistance, their yield performance must be carefully evaluated against elite conventional hybrids to ensure net agronomic benefit.

It is also imperative to note that while GMCs have been promoted for their potential to enhance agricultural productivity, the assumption that they inherently lead to higher yields is increasingly being challenged. Several field‐level assessments and meta‐analyses indicate that yield performance of GMCs is not uniformly superior to their conventional counterparts, particularly in resource‐constrained or rainfed farming systems.

For example, Bt cotton in India showed initial gains in yield due to effective pest control, but subsequent stagnation or decline in yield has been observed in many regions, often attributed to secondary pest outbreaks, soil fatigue, or inadequate agronomic support (Kranthi & Stone, 2020). Moreover, genetic modifications are typically targeted at specific traits such as pest resistance or herbicide tolerance, not directly at enhancing yield potential per se. This underscores a critical limitation: unless GMCs are bred into high‐yielding, well‐adapted genetic backgrounds and integrated with sustainable agronomic practices, their yield benefits may remain marginal or even regress over time. Therefore, a nuanced risk‐benefit analysis must consider not just the genetic potential of the GMC but also ecological interactions, socioeconomic variables, and long‐term field performance across agroecologies.

In some cases, GMCs may exhibit no yield advantage, or even lower productivity, when underlying stress conditions (such as drought, soil salinity, or pest composition) do not match the engineered traits, or when the crops face new biotic and abiotic challenges (Azadi et al., 2016). Furthermore, the “yield drag” effect observed in some GE lines (Gurian‐Sherman, 2009) possibly due to pleiotropic or insertional effects, emphasizes these concerns. This raises a critical imperative for policymakers, scientists, and stakeholders to rigorously weigh claimed yield benefits against observed performance variability, the economic realities for smallholder farmers, and the potential trade‐offs with crop diversity and resilience. Only through transparent, context‐specific risk‐benefit analysis, the deployment of GMCs can be justified, particularly in environments where food security depends on reliable and sustainable yield performance.

### 6.2 Presence of foreign DNA

The existence of alien DNA in GMCs is a major area of disagreement. Detractors argue that introducing foreign genes into crop genomes has the potential to have unforeseen consequences, including unfamiliar allergic reactions, environmental disturbances, or the production of new pests and diseases. A case in point is the StarLink corn fiasco in America, where a GM maize containing the Cry9C protein (approved only for animal feed) inadvertently entered the human food supply. This prompted product recalls due to concerns over possible allergenicity (Caradus, 2023). Ecological problems have also been documented; for instance, the widespread cultivation of Bt cotton, which had been GE to produce insecticidal proteins, reduced bollworm numbers at first but inadvertently created secondary infestations such as whiteflies and mirid bugs. This required additional pesticide sprays and disrupted pest control systems (Kranthi & Stone, 2020). These cases illustrate how the use of transgenic DNA can have complex ecological ripple effects that are inconsistent with environmentally sustainable farming practices.

### 6.3 Role of apex approval committees

The GEAC, functioning under the Ministry of Environment, Forest and Climate Change, serves as the apex body responsible for the approval of GMCs in India. Comprised of multidisciplinary experts, including specialists in molecular biology, ecology, toxicology, and food safety, the GEAC integrates perspectives from research institutions, government agencies, agricultural universities, and environmental non‐government organizations (Ahuja, 2018). This diversity aims to ensure that biosafety assessments reflect broad scientific understanding.

However, despite this framework, India's regulatory system for GMCs is often criticized as weak and plagued by conflicts of interest (GRAIN, 2023). Members of the GEAC are affiliated with institutions actively involved in transgenic research or development, leading to questions about impartiality in decision‐making. Furthermore, while the committee claims to align with international biosafety standards and draws on global regulatory guidance (Mahanayak, 2024), the actual enforcement, transparency, and independence of the regulatory process remain under scrutiny (Shukla et al., 2018).

These systemic issues raise concerns about whether environmental and health risks are adequately assessed before commercialization. Though the GEAC is positioned as India's most senior authority on the environmental evaluation of GMOs, its effectiveness is ultimately constrained by limited institutional autonomy and overlapping stakeholder interests (Shukla et al., 2018).

### 6.4 Biological, social, and legal concerns

Unintended release of GMCs through natural mechanisms such as cross‐pollination, seed dispersal, and volunteer plants represents a serious environmental and legal threat, particularly in countries like India. Indian agriculture is dominated by smallholder farmers with fragmented landholdings, where individual plots are small and often adjacent to others. This proximity increases the likelihood of unintentional gene flow, where pollen or seeds from a GM crop may travel into a neighboring non‐GM field. Such contamination can lead to situations where a farmer is involuntarily cultivating a GMC, even without consent or purchase, raising not only ethical and legal challenges but also forcing farmers into costly litigation (Yun et al., 2025).

A cautionary example is the well‐known Percy Schmeiser case in Canada, where GM canola appeared in a farmer's field despite never having been purchased, likely due to seed drift or cross‐pollination. The farmer was taken to court by Monsanto for patent infringement. Though the Canadian Supreme Court upheld Monsanto's patent, it found that Schmeiser did not owe damages as he had not benefited from the GM trait (Van Dooren, 2010). Nevertheless, the case highlighted the legal complexities and vulnerabilities faced by farmers.

In India, similar risks loom large, especially with crops like GM mustard, which is open‐pollinated and known to exhibit cross‐pollination rates between 12% and over 30%, depending on environmental factors and pollinator activity. The presence of weedy relatives such as *Brassica rapa* and other wild *Brassica* species in Indian agroecosystems increases the probability of gene flow to wild or feral populations. This could result in the emergence of herbicide‐resistant superweeds or unwanted introgression into native germplasm, threatening biodiversity, seed sovereignty, and potentially forcing farmers to accept GM traits even if they prefer to remain GM‐free (Shiva, 2019; Shiva et al., 1999).

Such unintentional spread raises urgent calls for robust biosafety regulations, clear liability frameworks, and effective coexistence strategies to protect both the environment and farmer rights. Without these safeguards, the release of GM crops may compromise ecological stability, undermining the very foundation of responsible biotechnology governance in India.

Ethical issues surrounding transgenic crops are multifaceted, involving concerns about food safety, environmental sustainability, and social justice. The dominance of multinational corporations over GM seed patents restricts farmer autonomy and fosters economic dependency, especially among smallholders. Issues of equity arise when access to transgenic technologies is uneven, favoring industrial agriculture over marginal communities. Furthermore, the lack of transparency in regulatory processes and limited public consultation undermines democratic governance, while inadequate labeling impairs consumer choice. Some communities also raise moral and religious objections to cross‐species gene transfer, which they view as unnatural. Finally, the widespread adoption of GM crops can displace traditional farming systems and erode indigenous knowledge, posing a threat to cultural and seed sovereignty.

To mitigate the environmental and legal risks associated with GMCs, alternative strategies must emphasize coexistence frameworks, robust containment, and farmer protection mechanisms. One key approach is the implementation of buffer zones and physical isolation distances to reduce cross‐pollination between GM and non‐GM crops. Additionally, the use of male sterility systems or chloroplast engineering (Chase, 2006), which restricts gene flow through pollen, can help prevent transgene escape. Establishing a clear liability regime that protects non‐GM farmers from unintended contamination and provides compensation for economic loss is equally critical. Moreover, investing in non‐transgenic breeding technologies, such as MAS (elaborated below), may offer safer and more publicly accepted alternatives for trait improvement without foreign gene introduction. These strategies, supported by transparent regulatory oversight and participatory decision‐making, can promote the safe, equitable deployment of crop biotechnology in India.

### 6.5 Alternative strategies

Cisgenesis, intragenesis, genome editing, and MAS and GS are other prominent alternatives to the conventional transgenic approach.

#### 6.5.1 Cisgenesis

While transgenesis involves taking genes from unrelated species, cisgenesis involves transferring genes from sexually compatible or the same species. This makes the new plants genetically closer to those produced by conventional breeding (Jacobsen & Schouten, 2008; Vasudevan et al., 2023). This closeness has led some scientists to propose that cisgenic crops be regulated differently than transgenic crops, potentially simplifying regulation while increasing consumer acceptance (Dudziak et al., 2019). A notable example is the development of a cisgenic potato evolved for resistance to late blight disease by introducing *Rpi*‐genes from wild potato relatives, without incorporating any foreign DNA (Holme et al., 2013). Similarly, the cisgenic Arctic Apple was developed to stop browning by blocking polyphenol oxidase genes from the apple itself, as a case of transgene‐free quality enhancement (Jogdand et al., 2017). Other than potential shorter breeding periods and avoidance of “linkage drag” associated with traditional backcrossing, cisgenesis is feasible for specific improvement with positive regulatory and ecological profiles. Using genes within the crop's own natural gene pool, cisgenesis reduces the threat of unwanted gene flow and can be better accepted by the public than conventional GM crops (Mwenje & Chimwamurombe, 2023). Hence, cisgenesis is a promising route to future crop improvement, which matches innovation with ecological and consumer requirements.

#### 6.5.2 Nanobiotechnology

Plant genetic engineering is extremely crucial in sustainable agriculture to enhance the quality of crops, productivity, and resistance to abiotic and biotic stress (Shaheen & Abed, 2018). Traditional plant genetic engineering techniques such as *Agrobacterium*‐mediated transformation, electroporation, biolistic bombardment, and polyethylene glycol‐mediated genetic transformation have drawbacks such as species dependency, non‐reproducibility of transformation efficiency, and high costs (Fiaz et al., 2021). In recent years, gene delivery strategies for plant genetic modification based on nanotechnology have been proposed (Wang et al., 2019; Zhang et al., 2019). This nanotechnology‐based approach has the prospect of excellent transformation efficiency, biocompatibility, excellent preservation of exterior nucleic acids, and the capacity for regeneration in plants. However, this nanoparticle‐mediated plant gene‐delivery system is nascent and has extremely large barriers for general use. Researchers are also exploring the integration of plant nanotechnology with CRISPR‐Cas‐directed genome editing (Naik et al., 2022). Nanomaterial‐delivered genetic transformation, conceptual developments, technological developments, and applied genetic transformation research are envisioned to propel plant genetic engineering in novel agriculture.

#### 6.5.3 Intragenesis

It is a genetic engineering approach that involves the insertion of genes or gene fragments derived from the same or closely related species, but in novel combinations not typically found in nature. Unlike transgenesis, which introduces foreign genes (often from unrelated species), intragenesis stays within the species' gene pool or compatible gene pools, addressing public concerns about “foreign DNA” in crops. Intragenic modifications can include regulatory elements (like promoters or terminators) and coding sequences rearranged to enhance gene expression, stress tolerance, or disease resistance. For example, in potato, intragenesis has been used to enhance resistance to fungal pathogens by using native promoters to drive the expression of endogenous resistance genes (Haverkort et al., 2016). This method holds promise for precise trait improvement while maintaining species integrity, potentially improving consumer acceptance compared to transgenic GMOs. Intragenesis is particularly useful for modifying traits such as delayed fruit ripening, enhanced nutrient profiles, and improved biotic stress resistance, all without introducing genes from distantly related organisms.

#### 6.5.4 CRISPR‐Cas genome editing

CRISPR is the foundation of the most widely used gene‐editing tool. It employs chemically made enzymes known as nucleases, which act like molecular scissors to cleave the DNA double helix, enabling the cell's own repair system to repair the break (Ghoshal, 2024). CRISPR technologies, due to their precision, bypass numerous adverse side effects of conventional plant breeding methods. CRISPR provides exact modifications to plant DNA without introducing foreign DNA. In addition, the regulators apply weaker regulations to these genome‐edited crops that are not transgenic since they contain DNA sequences that naturally exist (Ahmad et al., 2023). CRISPR is now employed to produce over 500 products in the world that are in varying stages of development ranging from basic research, late‐stage R&D, to near commercialization. The first CRISPR‐edited crop, the Sicilian Rouge High gamma‐aminobutyric acid (GABA) tomato, was commercially released by the Japanese company Sanatech Seed in 2021 (Waltz, 2021). The CRISPR‐edited tomato contains elevated levels of GABA, an amino acid/neurotransmitter found to lower blood pressure (Ezura, 2022). Conscious Greens, a GE green leafy vegetable variety with enhanced flavor and color, is slated for release in the US market in 2023 (Seong et al., 2024). The global market for genome‐edited products was around $5 billion in 2021 and is projected to reach up to $12 billion by 2026 (Parisi & Rodríguez‐Cerezo, 2021; Parisi et al., 2016).

The potential of CRISPR‐Cas genome editing has been well demonstrated in India's trailblazing success with the development and release of its first genome‐edited rice varieties—DRR Rice 100 (Kamala) and Pusa DST Rice 1. Launched on May 4, 2025, by Union Agriculture Minister Shri Shivraj Singh Chouhan, the achievement makes India the world's first country to approve genome‐edited rice for cultivation. Developed by the ICAR, these lines are a reflection of what can be done in providing long‐term answers to global agricultural issues. DRR Rice 100 (Kamala), a product developed by ICAR‐IIRR from popular parent Samba Mahsuri, has enhanced drought, salinity, and climate stress tolerance. This crop gives a 19% increase in yield, 20% greenhouse gas emission reduction, and saves 7500 million m^3^ of irrigation water, a giant leap toward climate‐smart agriculture. Alternatively, Pusa DST Rice 1, developed by ICAR‐IARI, gives 9.66%–30.4% higher yields in saline and alkaline soils, and has the potential to add 20% to rice production in unfavorable environments (Rao & Chandrasena, 2025).

#### 6.5.5 Marker‐assisted selection and genomic selection

MAS and GS are powerful tools in modern plant breeding that accelerate the development of improved crop varieties without altering the genome through external DNA insertion (Boopathi, 2020). MAS uses DNA markers linked to specific traits (such as disease resistance, drought tolerance, or yield) to select desirable genotypes early in the breeding cycle. It is particularly effective for traits governed by a few major genes. GS, on the other hand, uses genome‐wide markers and statistical models to predict breeding values for complex, quantitative traits, making it ideal for polygenic traits like grain yield, drought resilience, and nutrient use efficiency.

Compared to transformation techniques, MAS and GS do not involve direct manipulation of DNA and hence are often more acceptable to regulatory bodies and the public. Transformation enables the direct introduction or editing of specific genes, which can be powerful when native genetic variation is absent or insufficient. While transformation allows for precise trait introduction, MAS and GS leverage natural or existing genetic diversity within the species, making them cost‐effective and faster for routine breeding.

Together, these tools form a complementary toolkit for plant improvement, where MAS and GS are used for enhancing breeding efficiency, and transformation is applied when novel traits or genes are needed that are not accessible through conventional breeding.

### 6.6 Agroecological approaches to sustainable agriculture

Agroecology centers on sustainable agriculture by integrating biodiversity, soil health, and ecological stability within farm systems. By using methods such as multi‐year crop rotation (for example, rotating legumes with cereals to replenish nitrogen naturally), polycultures (such as planting maize and beans together to save space and pest susceptibility), and biological pest control (such as using predatory insects such as ladybugs to suppress aphids), agroecology aims to minimize dependence on GMCs and chemical inputs. This approach employs a genetic diversity that already exists in locally adapted, traditional crops and increases resistance to pests, disease, and climatic uncertainty. Maintaining agroecosystem diversity not only preserves ecological integrity but also human well‐being and cultural heritage linked with indigenous farming. In line with this, the promotion of agroecological practices is guided by a precautionary, long‐term approach that achieves maximum environmental sustainability, food sovereignty, and resilience amid global challenges.

### 6.7 World approval and adoption environment of GMCs

So far, 46 countries in the world are pursuing active research on GMCs (International Service for the Acquisition of Agri‐biotech Applications’ GM Approval Database). The majority of GMC approval trends over recent years have been focused on addressing the looming food crisis, which impacts 281.6 million people in 59 nations (https://www.fsinplatform.org/grfc2024). The European Commission (EC) has approved GM maize and re‐approved the use of GM oilseed rape for animal feed and food (EU governments fail to agree on gene‐editing rules despite patent exception). Africa's Development Agenda 2063 aims to overcome the food crisis by adopting GM‐dependent agriculture (Catherine et al., 2024). Meanwhile, China approved early in 2024 to import and sow more kinds of GM soybeans and corn, as part of a strategic initiative to boost food security (https://www.business‐standard.com/industry/agriculture/china‐expands‐imports‐planting‐approvals‐of‐additional‐gene‐modified‐crops‐124011800505_1.html). There is sharp opposition to the EC's New Genomic Techniques law in a move to categorize certain GMOs as equal to crop varieties developed through conventional breeding. With Hungary, which has a constitutional ban on the use of GMOs in agriculture, in line for the next 6‐month presidency, the revocation of embargos on such GMC approvals may prove challenging.

As of October 2024, over 30 countries have approved the cultivation of GM crops, pointing to the increasing reliance on biotechnology in addressing pressing challenges such as food security and global climate change (Source: https://www.isaaa.org/gmapprovaldatabase/). In 2019, 29 countries were cultivating GMCs around the world, rising to 32 by 2024, with an additional three African countries among the adopters. Ghana was the newest country to approve GMC cultivation, granting commercial approval in 2024 for GM pod‐borer‐resistant cowpea, developed by the Council for Scientific and Industrial Research‐Savanna Agricultural Research Institute (CSIR‐SARI), Nyankpala, Ghana.

There was a combined total of 614 GMC approvals included in the Global Database for Genetically Modified Approvals as of August 2024. Of them, maize garnered the largest number of approvals (290 events), followed by cotton (72 events) and potato (52 events). The majority of them (405 events) involved stacked trait events, with the rest 209 being for single traits. The year 2022 saw the highest approvals in food, feed, and cultivation categories. Between 1998 and 2023, Colombia led food‐related GM approvals, followed by the European Union and Argentina in feed and cultivation approvals, respectively. These are pointers toward the steady global advancement of GMC adoption as countries persist in using biotechnology in cultivating agricultural productivity and sustainability (Source: https://www.isaaa.org/gmapprovaldatabase/).

## 7 BIOFORTIFICATION: TRANSGENIC AND CONVENTIONAL APPROACHES

A balanced diet is indeed important for overall human health and well‐being. Traditional meals often consist of a variety of foods that provide essential nutrients like proteins, carbohydrates, fats, vitamins, and minerals. Consumption of diverse foods guarantees the intake of necessary nutrients for the body to function well, resulting in a healthy weight and avoidance of chronic diseases.

Biofortification is the production of crops by breeding to enhance their nutrient levels, that is, the quantity of essential vitamins and minerals in their consumable parts. While genetic engineering can be employed as a biofortification means, it is not exclusive. Conventional breeding techniques like hybridization and selective breeding have been employed for decades to increase the nutritional content of crops, with a number of biofortified hybrids and varieties having already been released commercially (https://pib.gov.in/PressReleaseIframePage.aspx?PRID=1984070). Global efforts, such as HarvestPlus, have increased vitamin and mineral concentration in crops by leaps and bounds, employing conventional breeding technologies without going down the GE route (https://www.harvestplus.org/harvestplus‐biofortified‐crops‐map‐and‐table‐updated‐with‐2020‐data/).

However, there are benefits to GE technology in terms of accuracy and efficiency in order to attain the desired nutritional attributes in plants. To illustrate, biofortified GE crops have been GM to address specific nutritional needs of populations, for instance, Golden Rice, which has been engineered to produce beta‐carotene, a vitamin A precursor. Ultimately, the choice to utilize GE technology for biofortification must be carefully considered with regard to matters of safety, regulatory approval, social acceptability, and expense. It is crucial to explore multiple approaches, ranging from classical breeding to innovative biotechnology, to ensure that biofortified crops are found and effective for those in need. A special emphasis on balanced foods, being inherently safe to the economy, environment, agriculture, and humanity, can be an extra measure to mitigate risks from emerging technologies (Lee & Natesan, 2006).

## 8 PROBLEMS CONCERNING GMCS

**FIGURE 5:**
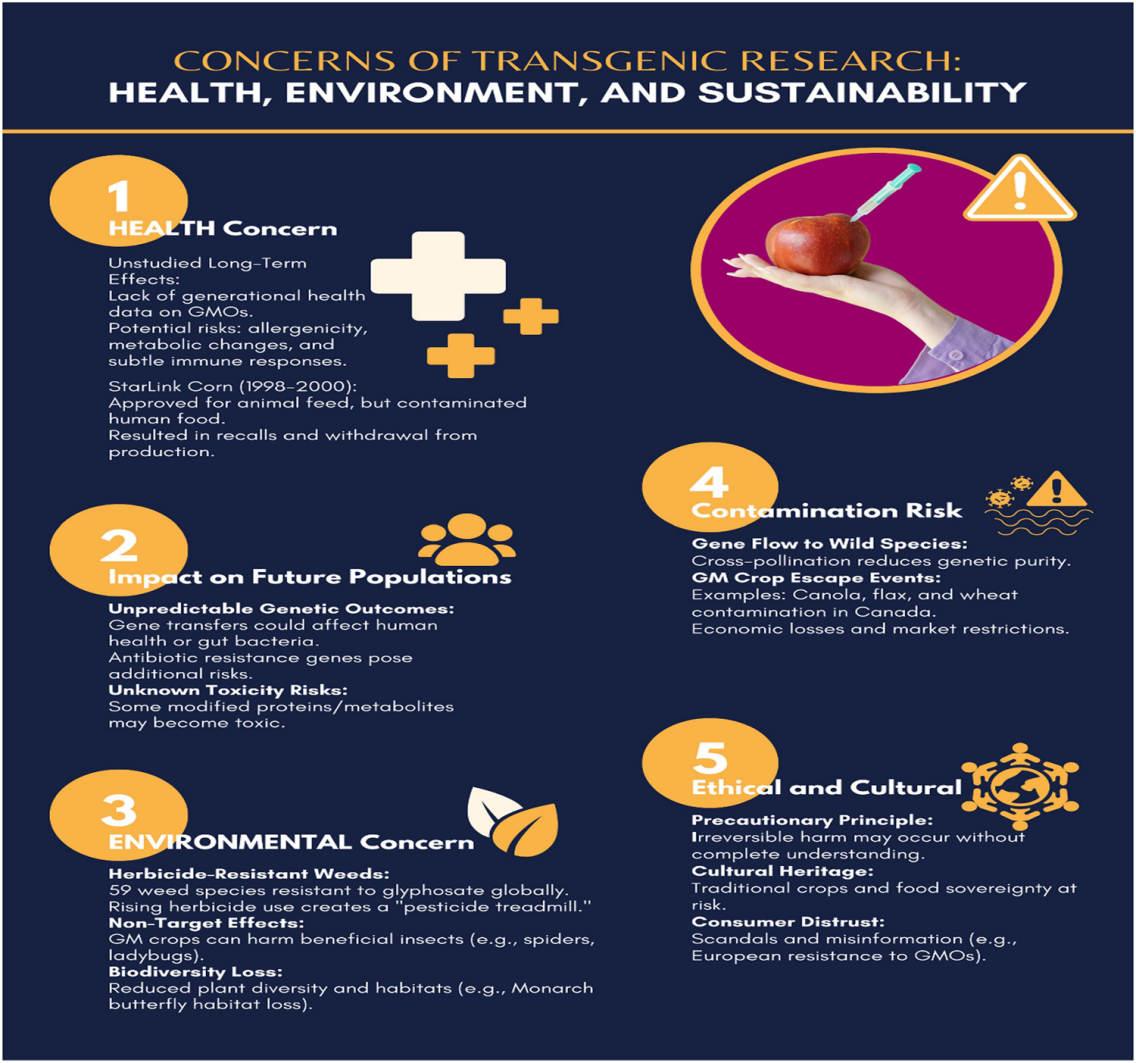
Concerns of transgenic research related to health, environment, and sustainability.

Concerns regarding the long‐term consequences of transgenic proteins and the erosion of genetic diversity must be addressed. Modified proteins in GMCs are able to have unexpected and unpredictable consequences on other co‐expressed proteins (Figure 5).

While India has an established framework for transgenic crops under the Rules, 1989 of the Environment (Protection) Act, the regulatory stance toward emerging gene‐editing technologies—particularly CRISPR/Cas‐based approaches—remains evolving. In 2022, the Government of India issued guidelines exempting site‐directed nuclease (SDN)‐1 and SDN‐2 gene‐editing applications (which do not involve foreign DNA insertion) from the stringent regulations applied to GMOs, aligning with the principles of conventional mutagenesis. However, ambiguity persists regarding the classification, field testing, and commercialization of genome‐edited crops that closely resemble conventionally bred varieties.

Public sentiment in India toward GM technologies remains cautious, influenced by concerns over biosafety, environmental impacts, and corporate control of seed systems. Nevertheless, there is growing acceptance among scientists and policymakers of genome editing as a safer, more precise, and publicly acceptable alternative to traditional transgenics. A clearer, science‐based regulatory framework, supported by transparent communication and stakeholder engagement, will be crucial for advancing the responsible adoption of CRISPR‐based innovations in Indian agriculture.

### 8.1 Unsettled health impacts

As GMCs are quite recent, much data on long‐term impacts of transgenic protein consumption over generations in humans and animals are still lacking. Future unforeseen risks, such as subtle immune effects, allergenicity, or metabolic issues, can occur. Because these proteins are not natural in the human diet, health evaluations for long‐term implications are crucial.

While India's regulatory framework provides structured guidelines (http://geacindia.gov.in/guidelines‐and‐protocols.aspx) for assessing health impacts of GM crops, including allergenicity, toxicity, and compositional equivalence, critical gaps remain in long‐term studies, post‐market surveillance, transparency, and tailored risk communication. Addressing these (Table 1) will strengthen public confidence and align with international best practices mandated by the GEAC's risk analysis. Public reports often focus on economic or environmental debates, while transparent sharing of health‐safety data, biosafety trial protocols, and expert interpretations is limited. Further, absence of user‐friendly platforms summarizing allergenicity and toxicity outcomes reduces consumer trust and informed uptake.

**TABLE 1:** Identified health impact components that are missing in risk assessment of genetically modified crop (GMC) in India.

Identified risks/issues	How to rectify/fill the gap?
Substantial equivalence analysis	A GMC's composition (e.g., proteins, nutrients, anti‐nutrients) should be compared with its non‐GM counterpart to identify unintended changes. Any nutritional imbalance or novel compound triggers deeper investigation.
Allergenicity evaluation	Given the potential introduction of foreign proteins (e.g., Brazil‐nut gene in soybean), assessment requires: Sequence homology with known allergens, Digestibility in simulated gastric fluids, Serological testing using sera from allergic individuals.
Toxicological assessment	GMC evaluation should include: Acute, sub‐chronic, or chronic toxicity testing in appropriate animal models, Examination of biochemical, hematological, immunological, and organ‐specific markers, Carcinogenicity or reproductive endpoints if warranted
Metabolic and gene interaction analysis	Unexpected metabolic changes may arise from gene insertion, causing expression shifts that necessitate metabolomic profiling and targeted toxicity tests. Hence, data on such interaction should made available.
Chronic health impacts	Institute long‑term feeding trials with relevant livestock or rodent models, conducted under GLP standards.
Post‑approval health monitoring	Set up a national monitoring registry for reporting adverse reactions or allergenicity cases among consumers.
Transparency and communication	Publicly release safety assessment dossiers, especially allergenicity/toxicity data via GEAC website or Biosafety Clearing House portals
Integration of toxicology with environmental stress	Study combined effects of GM ingestion under pesticide exposure or nutrient imbalance conditions.
Vulnerable populations	Evaluate health risks specifically in infants, pregnant women, and immunocompromised groups using sub‑population dietary models.

Abbreviations: GEAC, Genetic Engineering Appraisal Committee; GLP, Good Laboratory Practice; GM, genetically modified.

#### 8.1.1 Fact sheet—Case 1

*StarLink Corn: A cautionary tale*: The US Environmental Protection Agency (EPA) licensed StarLink, a GM yellow corn crop by Aventis Crop Science (later acquired by Syngenta), in 1998 (Bucchini & Goldman, 2002). StarLink was designed to express the *Cry9C*, which encodes a Bt toxin lethal to pests, including the European corn borer. But the toxin was of concern since it was heat‐resistant and degraded very slowly in the human gastrointestinal tract, giving way to the problem of allergenicity. As studies on its possible allergenicity were indefinite, the EPA restricted StarLink corn for animal feeding only, prohibiting its use in human foods (Bucchini & Goldman, 2002). It was against this regulation that the biological nature of corn and the complexity of the US crop‐handling system made contamination difficult to prevent. In September 2000, StarLink corn, approved only for animal feed and industrial use, was noticed in taco shells and other food products intended for human consumption, violating its restricted approval. This incident triggered widespread food recalls and public concern over the regulation of GMOs. As a result, Aventis, the developer of StarLink, voluntarily withdrew its registration, and the cultivation of this variety was discontinued. Despite the controversy, other Bt corn varieties, engineered to express alternative pesticidal proteins, have undergone rigorous safety evaluations for toxicity and allergenicity. The US FDA, which oversees the safety of GM foods, has affirmed that these GM corn varieties are safe for human consumption and nutritionally equivalent to their conventional counterparts. Today, they are widely cultivated across millions of acres in the United States.

### 8.2 Potential negative effects

The long‐term health implications of sustained consumption of food derived from GMCs, particularly across generations, remain insufficiently understood. This uncertainty underscores the necessity of precautionary evaluation, as widespread adverse health outcomes, once embedded in a population, may be irreversible or difficult to mitigate. Genetically induced modifications in host metabolism may inadvertently trigger or deregulate endogenous biochemical pathways, potentially leading to the synthesis of novel toxic metabolites or the accumulation of naturally occurring toxins at harmful levels. Consequently, comprehensive health safety assessments for each GMO are essential and mandated by regulatory bodies such as the European Food Safety Authority (EFSA Panel on GMOs, 2011).

A central concern in the biosafety dissertation is the potential for horizontal gene transfer (HGT), the movement of transgenic DNA from GM crops into human somatic cells or gut microbiota. This risk becomes especially critical when transgenes encode antibiotic resistance markers, which were historically used during GMO development. If such genes were to integrate into commensal or pathogenic microbial populations, it could compromise the efficacy of clinically important antibiotics and exacerbate antimicrobial resistance (Philips et al., 2022). While the probability of HGT is considered low under natural gastrointestinal conditions, both the Food and Agriculture Organization and the World Health Organization advocate for the exclusion of antibiotic resistance genes in commercialized GM crops as a precautionary measure.

### 8.3 Loss of crop genetic diversity and environmental issues

Crop germplasm collections hold vast genetic diversity, which is needed for the solution to issues such as drought, pests, and diseases. Crop genetic diversity is the secret of agricultural resilience through a sufficient range of options that can be employed in adaptation to changing situations and future issues. Genetic profiling and phenotyping technology advances have brought forth promising paths for the comprehensive characterization of high germplasm, hence opening untapped diversity to be applied in plant breeding operations. Conversely, excessive reliance on a limited number of GMCs can increase vulnerability to pests or diseases attacking those specific crops because genetic homogeneity will lower general resilience (Philips et al., 2022). Stressing and leveraging natural genetic variability can provide a buffer against such threats and ensure the resilience of our food supplies. GM technology has often led to increased herbicide use (e.g., glyphosate), which impacts soil quality and biodiversity in the long term (Schütte et al., 2017). These compounds have the potential to accumulate within ecosystems over time, poisoning soil microorganisms, pollinators, and non‐target plant life. Some GM crops can grow in ways that harm other non‐target species and water and soil ecosystems. Herbicide‐tolerant crops have led to more herbicide use, which in turn reduces plant diversity and degrades habitats for other species.

For instance, the Monarch butterfly population has declined by over 90% in less than two decades, largely due to the widespread use of glyphosate herbicide, which eradicates milkweed, the primary breeding ground and food source for Monarch larvae, resulting in the loss of more than 165 million acres of critical habitat (Hansen Jesse & Obrycki, 2000). On the other hand, it has been shown through subsequent studies that crops engineered to express Bt toxins have generally demonstrated minimal risk to non‐target insect species, including beneficial organisms such as pollinators and natural predators (Gatehouse et al., 2002).

In addition to terrestrial impacts, GMCs such as Bt corn have been linked to ecological disturbances in aquatic ecosystems. Studies have detected Bt toxins and crop residues in nearby streams, which have shown adverse effects on caddisflies, which are aquatic insects closely related to lepidopteran pests targeted by Bt. These effects include increased mortality and impaired development (Rosi‐Marshall et al., 2007). Such ecological disruptions, both terrestrial and aquatic, raise serious concerns about the broader implications of transgenic crop cultivation on long‐term agricultural sustainability (Schütte et al., 2017).

However, soon after such claims, several discussions about GM crops have become highly controversial, where even scientific studies are often caught in fierce arguments (Waltz, 2009). It explains that instead of focusing just on facts, debates often turn into fights between opposing beliefs and opinions.

Under this situation, what's essential for the general public is:

1. *Access to clear, unbiased information*: People need science‐based facts explained in simple language, free from political or corporate influence.
2. *Transparency from regulators and scientists*: Public trust grows when decision‐making processes, research data, and potential risks are openly shared.
3. *Open dialogue and education*: Encouraging informed discussions, not just debates, helps people understand both the benefits and limitations of GMOs.
4. *Inclusion of public concerns in policy*: Listening to farmers, consumers, and civil society ensures that decisions are not just scientifically sound but also socially accepted.

This builds trust and helps society make balanced choices in the face of complex technologies.

### 8.4 Gene flow to wild and weedy relatives and loss of biodiversity

Gene flow from GM crops to their weedy and wild relatives is a very challenging problem in terms of unwanted genetic pollution, particularly among highly related crop species. For example, gene exchange between GM rice and wild or weedy rice populations such as *Oryza rufipogon* and *Oryza nivara* has been shown to increase the potential for generating “superweeds” that are herbicide‐resistant or more competitive, thereby destabilizing agroecosystems and complicating weed control (Lu & Yang, 2009). Similar cases in GM mustard (Groot et al., 2003) have already been indicated in the above sections. Such unanticipated gene flow compromises the genetic integrity of wild gene pools, rendering them less useful as reservoirs of genes for future crop enhancement, for example, disease resistance and climate adaptability. Incidents like the StarLink maize case in the United States, as mentioned above, underscore the pragmatic challenges in avoiding genetic contamination even under a regulated regime. To address these risks, most countries have adopted coexistence practices like buffer strips and isolation distances to reduce gene flow between GM and non‐GM crops (Trkulja et al., 2014). But early warnings from environmentalists about the ecological and agronomic effects of growing GM crops are increasingly being proved correct as these issues take root in real‐world contexts.

### 8.5 Insect‐resistant crops and conjugated pesticide use

GM insect‐resistant Bt crops contain a toxin that is toxic to particular insect pests. Genetic modification tends to produce unforeseen impacts on the modified organism as well as the environment. Research indicates that Bt toxins affect non‐target organisms as well. Such “non‐target effects” can cause the organism to alter in unforeseen ways other than the intended gene modification (Devos et al., 2016). Predatory insects such as spiders, wasps, ladybugs, and lacewings, which feed on insect pests poisoned by Bt toxins, have been affected by a negative effect after consuming prey that ingested these toxins (Naranjo, 2009). This also raises issues over the overall ecological effect of Bt crops on populations of beneficial insects. Despite the initial decrease in external application of insecticide with the introduction of Bt corn, a great deal of the insecticidal endotoxin is emitted by the GM plant itself. Insecticide use is now rising as targeted insects adapt to become resistant to Bt toxins. For instance, the number of pesticides (herbicides, insecticides, and fungicides) applied to cornfields in North America increased from approximately four pesticides per hectare in 1996, when GM crops started being used, to over 13 pesticides in 2023 (Cernicky, 2023).

### 8.6 Increased herbicide application and emergence of herbicide‐resistant superweeds

The continuous re‐planting of GM herbicide‐resistant varieties has also triggered a dramatic escalation of herbicide application. Herbicide sales in Canada rose by 244% from 1994 to 2021 (Werner et al., 2022). The growing dependency on chemical weed control poses challenges to long‐term agricultural sustainability because resistant weeds continue to spread, necessitating more or different herbicide formulations.

Widespread application of some herbicides in conjunction with GM herbicide‐resistant crops has resulted in the emergence and dissemination of herbicide‐resistant weeds, commonly known as “superweeds.” They evolve through repeated exposure to the same herbicide, thus posing difficulties to conventional weed control (V. Kumar et al., 2008). Since 1996, when herbicide‐resistant GMCs were released, at least 59 species of weeds have become resistant to glyphosate, one of the most widely applied herbicides worldwide. Resistance to herbicides predated GMCs, but the release of herbicide‐resistant GMCs, such as glyphosate‐resistant “Roundup Ready” crops, has intensified the problem. Globally, 59 weed species are resistant to glyphosate, eight in Canada, and 18 in the United States. This resistance also poses serious economic issues to farmers, with controlling weeds in infested fields costing 50%–100% extra in herbicide costs per hectare. Certain weeds have even become resistant to more than one herbicide, making control even more challenging and leading to more overall herbicide use in what has been called the “pesticide treadmill.” To counter this, biotech corporations have created GMCs with more than one herbicide‐resistant trait, allowing for additional herbicides to be used (Owen & Zelaya, 2005; Schütte et al., 2017). For instance, Canada approved GM corn with resistance to 2,4‐D in 2012, dicamba in 2016, and the two herbicides in 2020. It has been reported that 80% of Canadian GM corn became resistant to over one herbicide by 2023 (Daggy et al., 2024).

### 8.7 Emergence of super‐pests

Over time, certain insect pests have developed resistance to toxins from GE insect‐resistant crops, for example, Bt crops. The first reported instance in Canada occurred in May 2019, when the European corn borer had grown resistant to a GM trait specifically developed to kill it. The first publicly reported instance anywhere in the world (Sappington, 2024), there have also been reports of resistance elsewhere in other countries where insect pests that have been the target of Bt traits in crops like corn and cotton have adapted to survive. United States, South Africa, and Brazil reports indicate that resistance is increasingly becoming a general issue (James, 2011). A 2023 paper documented that as of 2020, there were 26 instances of Bt resistance in seven countries infesting 11 species of insects versus three reported instances back in 2005 (Razzaq et al., 2023). In addition, the early signs of resistance in other species reveal that this issue is still evolving, raising questions about the long‐term efficacy of Bt crops in controlling pests.

### 8.8 Contamination risks

Contamination due to GMC has grave environmental, economic, and social impacts.

#### 8.8.1 Fact sheet—Case 2

*Unintended escape events in Canada*: There have been several incidents of accidental escape of GMOs in Canada, including canola, flax, wheat, and pigs (McHughen, 2002). Although there were cases involving licensed GMCs, for example, canola and flax, some resulted from experimental manipulation in wheat and pigs. Some occurrences were singular (e.g., wheat and pigs), whereas others, like canola and flax contamination, have been recurring and steady. GMCs are prevalent in Canadian agribusiness, like canola, corn, soybeans, and white sugar beet, with limited GM alfalfa production in the eastern provinces. Commercialization of GM alfalfa is a matter of concern, particularly for organic production, as the risk of cross‐pollination undermines organic certification integrity. Prevention and regulation of such contamination remain a significant burden to Canadian farmers and policymakers (Macdonald, 2014).

### 8.9 Ethical considerations and responsibility toward future generations

Due to a lack of complete knowledge of GMO's long‐term consequences, there is a risk of inadvertently compromising future generations. The precautionary principle, favoring avoidance of behavior possibly having irreversible negative consequences, argues against the widespread application of technologies with unknown long‐term consequences. The majority of societies everywhere rely on locally adapted traditional crops, and preserving these traditional varieties, as opposed to replacing them with GMOs, is important to help maintain cultural heritage and provide people with ownership of their food, hence enabling food sovereignty (Kole et al., 2025).

## 9 CONSUMER CONCERNS REGARDING GM FOOD PRODUCTS IN OTHER COUNTRIES

Since their introduction in the 1990s, GM foods have remained a focal point of contention between consumers and regulators, especially in European markets. Consumers widely protest the necessity for GM foods, often asking, “Why should I eat them?” Interestingly, biotechnology is more readily accepted within the medical field, where individuals unequivocally value its health contributions in the present. Initial GM foods introduced in Europe bore primarily economic advantages for manufacturers above consumer profit, which contributed to public mistrust. Moreover, public trust in food safety has been eroded primarily through earlier fiascos, such as “mad cow disease” and poultry contamination by dioxin. Although not connected with GM foods, these incidents heightened the level of mistrust of official food safety assurances among the public. Corporate interests, disinformation, and perceived regulatory failure have also been responsible for cynicism. To alleviate consumer anxiety, the European Union has introduced GM food and animal feed compulsory labeling. Although there has been some gradual integration in recent years, very large proportions of the European public remain cynical about GMOs. In France, Germany, Italy, Poland, Spain, Sweden, and the Czech Republic, over one‐third of their population remains worried about it. But there is a disconnect between what the government practices and what the population believes: although 16 out of the 27 members of the EU have prohibited GMOs from being cultivated, Europe still imports an additional 118 GM foods. This discrepancy attests to the ongoing debate concerning GMOs in the food supply.

## 10 FUTURE PERSPECTIVES

Molecular breeding techniques, including MAS, GS, and gene editing (such as CRISPR, but excluding where extraneous DNA is introduced), offer powerful tools for addressing problems limiting global crop production and food security objectives without transgenic plants. These technologies utilize existing crop diversity and generally do not carry the same environmental concerns associated with conventional GM crops. GMC technology has raised significant benefits as well as unprecedented problems. While offering solutions to agricultural productivity, its unforeseen effects call for the imperative necessity of cautious management and continuous research into alternative and sustainable plant breeding practices. To ensure science‐based, transparent, and socially acceptable adoption of GM crops in India, policy reforms must bridge current regulatory gaps, and they are highlighted hereunder. Integrating biosafety science, public participation, and institutional accountability is critical for future approvals and public trust.

### 10.1 Critical policy considerations for GMCs in India

#### 10.1.1 Long‐term health and environmental risk assessment

The current regulatory approach in India focuses mainly on short‐term studies, such as 90‐day feeding trials, to evaluate the safety of GM crops. However, these are insufficient to detect long‐term or multi‐generational effects. Policymaking should mandate long‐term toxicology assessments, chronic exposure studies, and metabolic and allergenicity profiling using population‐specific data. Additionally, comprehensive ecological impact studies are necessary to ensure biosafety at the ecosystem level.

#### 10.1.2 Lack of post‐market surveillance

India lacks a structured program to monitor GM crops after their release into the market. There is no system in place to track adverse health effects, environmental consequences, or unintended socioeconomic impacts. A national post‐market surveillance framework should be established under ICMR (Indian Council of Medical Research) or FSSAI (Food Safety and Standards Authority of India) to monitor the health effects of GM foods and crops, ensuring a feedback loop into the regulatory process.

#### 10.1.3 Transparency in regulatory decision‐making

The decision‐making process for GM crop approval often lacks transparency. Public access to safety dossiers, field trial data, and meeting minutes of regulatory committees like GEAC is limited. To build public trust, these documents should be made publicly available and subjected to independent scientific scrutiny and peer review before final approval is granted.

#### 10.1.4 Independent, public‐sector testing

Current assessments rely heavily on data generated by private developers. This raises concerns about data integrity and conflict of interest. Strengthening public‐sector institutions such as ICAR‐IARI, SAUs, and DBT‐affiliated laboratories to independently validate biosafety, efficacy, and environmental impact claims is critical for objective decision‐making.

#### 10.1.5 Stakeholder consultation and social license

There is minimal inclusion of farmers, local communities, and civil society organizations in the GM crop approval process. Policy should institutionalize stakeholder consultations, state‐level inputs, and social impact assessments to align technological interventions with local needs, socio‐cultural values, and ethical concerns.

#### 10.1.6 Clear guidelines for coexistence and labeling

India currently lacks a coherent policy to manage the coexistence of GM, organic, and conventional farming systems. A robust framework should be developed to define buffer zones, enforce pollen containment measures, and mandate clear labeling of GM products to ensure consumer choice and traceability.

#### 10.1.7 Illegal and unregulated cultivation

There is widespread illegal cultivation of unapproved GM crops such as herbicide‐tolerant Bt cotton, posing biosafety and legal risks. State‐level biosafety committees and district authorities should be empowered and resourced to monitor fields, penalize offenders, and trace unauthorized seed supply chains.

#### 10.1.8 Evolving technologies not yet regulated

India's current biosafety guidelines are not fully equipped to regulate emerging technologies in CRISPR‐based base editing or RNAi crops. A forward‐looking policy must incorporate advanced editing frameworks in alignment with global best practices while also ensuring biosafety and public transparency.

#### 10.1.9 Inter‐ministerial coordination

GM crop governance in India is fragmented across multiple ministries and departments, leading to delayed decisions and policy paralysis. A central coordinating authority, such as a National Biotechnology Coordination Council, should be instituted to harmonize regulatory roles and facilitate streamlined, science‐based approvals.

#### 10.1.10 Education and awareness

Farmers, agricultural extension personnel, and consumers often lack accurate information about GM crops and their safe usage. Public awareness campaigns should be launched in regional languages, utilizing mass media and grassroots channels, to improve understanding, dispel myths, and promote responsible stewardship of GM technologies.

## 11 CONCLUSION

The future of sustainable farming in India and the world at large hinges on the judicious integration of diverse breeding strategies, transcending the naive polemic of GMOs. While the performance of GM crops like Bt cotton in India has demonstrated significant agricultural and economic benefits, the widespread adoption of such technology remains constrained by valid concerns regarding long‐term environmental impacts, pest and weed resistance, and significant socioeconomic implications for small‐scale farmers. India's bioregulatory regime, while evolving, has had a risk‐averse approach traditionally, as the Bt brinjal moratorium illustrates, with an interplay of subtle scientific assessment, public outcry, and political determination. Therefore, in order to properly secure the nation's food future, India requires a robust, transparent, and accountable regulation policy that not only ensures rigorous biosafety assessments but also promotes public trust, addresses socioeconomic inequalities, and judiciously focuses on biotechnological innovations like cutting‐edge genome editing and agroecological approaches that are aligned with the nation's specific agricultural landscape and sustainability agenda.

## AUTHOR CONTRIBUTIONS

**Chittaranjan Kole**: Conceptualization; formal analysis; methodology; resources; supervision; writing—original draft; writing—review and editing. **Sarita Pandey**: Investigation; methodology; writing—original draft; writing—review and editing. **Jeshima Khan Yasin**: Investigation; methodology; validation; visualization; writing—original draft; writing—review and editing. **Sujan Mamidi**: Visualization; writing—original draft; writing—review and editing. **Abhishek Bohra**: Visualization; writing—original draft; writing—review and editing. **Poulami Bhattacharya**: Writing—original draft; writing—review and editing. **Devraj Dhanraj**: Writing—original draft; writing—review and editing. **Gnanasekaran Madhavan**: Writing—original draft; writing—review and editing. **Dinesh Saini**: Writing—original draft; writing—review and editing. **Sayak Ganguli**: Writing—original draft; writing—review and editing. **Bhargavi HA**: Writing—original draft. **Sayanti Mandal**: Writing—original draft; writing—review and editing. **Sangita Agarwal**: Writing—original draft; writing—review and editing. **Arumugam Pillai M**: Writing—original draft; writing—review and editing. **Madhugiri Nageswara‐Rao**: Writing—original draft; writing—review and editing. **Swarup K Chakrabarti**: Writing—original draft; writing—review and editing. **Prakash C. Sharma**: Writing—original draft; writing—review and editing. **Akshay K. Talukdar**: Writing—original draft; writing—review and editing. **Jogeswar Panigrahi**: Writing—original draft; writing—review and editing. **Manikanda Boopathi N**: Conceptualization; investigation; methodology; supervision; writing—original draft; writing—review and editing.

## CONFLICT OF INTEREST STATEMENT

The authors declare no conflicts of interest.
